# Activin A and BMP4 Signaling Expands Potency of Mouse Embryonic Stem Cells in Serum-Free Media

**DOI:** 10.1016/j.stemcr.2020.01.004

**Published:** 2020-02-06

**Authors:** Baojiang Wu, Lin Li, Bojiang Li, Junpeng Gao, Yanglin Chen, Mengyi Wei, Zhiqing Yang, Baojing Zhang, Shudong Li, Kexin Li, Changshan Wang, M. Azim Surani, Xihe Li, Fuchou Tang, Siqin Bao

**Affiliations:** 1 The State Key Laboratory of Reproductive Regulation and Breeding of Grassland Livestock, Inner Mongolia University, Hohhot 010070, China; 2 Research Center for Animal Genetic Resources of Mongolia Plateau, College of Life Sciences, Inner Mongolia University, Hohhot 010070, China; 3 Guangdong Provincial Key Laboratory of Proteomics, Department of Pathophysiology, School of Basic Medical Sciences, Southern Medical University, Guangzhou 510515, China; 4 Beijing Advanced Innovation Center for Genomics and Biomedical Pioneering Innovation Center, College of Life Sciences, Peking University, Beijing 100871, China; 5 College of Animal Science and Veterinary Medicine, Shenyang Agricultural University, Shenyang 110866, China; 6 Cancer Research UK and Medical Research Council Oxford Institute for Radiation Oncology, Department of Oncology, University of Oxford, Oxford OX3 7DQ, UK; 7 Wellcome Trust Cancer Research UK Gurdon Institute, University of Cambridge, Tennis Court Road, Cambridge CB2 1QN, UK; 8 Inner Mongolia Saikexing Institute of Breeding and Reproductive Biotechnology in Domestic Animal, Huhhot 011517, China; 9 Peking–Tsinghua Center for Life Sciences, Peking University, Beijing 100871, China; 10 Ministry of Education Key Laboratory of Cell Proliferation and Differentiation, Beijing 100871, China

**Keywords:** mouse, embryonic stem cells, epigenetics, hypermethylation, development, chemically defined, genomic imprinting, blastocyst, pluripotency, implantation

## Abstract

Inhibitors of Mek1/2 and Gsk3β, known as 2i, and, together with leukemia inhibitory factor, enhance the derivation of embryonic stem cells (ESCs) and promote ground-state pluripotency (2i/L-ESCs). However, recent reports show that prolonged Mek1/2 suppression impairs developmental potential of ESCs, and is rescued by serum (S/L-ESCs). Here, we show that culturing ESCs in Activin A and BMP4, and in the absence of MEK1/2 inhibitor (ABC/L medium), establishes advanced stem cells derived from ESCs (esASCs). We demonstrate that esASCs contributed to germline lineages, full-term chimeras and generated esASC-derived mice by tetraploid complementation. We show that, in contrast to 2i/L-ESCs, esASCs display distinct molecular signatures and a stable hypermethylated epigenome, which is reversible and similar to serum-cultured ESCs. Importantly, we also derived novel ASCs (blASCs) from blastocysts in ABC/L medium. Our results provide insights into the derivation of novel ESCs with DNA hypermethylation from blastocysts in chemically defined medium.

## Highlights

• Activin A and BMP4 expand potency of mouse ESCs
• ASCs are hypermethylated and with stable genomic imprints
• ASCs developmentally closed to E4.5–E6.5 *in vivo* epiblast
• Hypermethylated ASCs directly derived from blastocyst by ABC/L medium

## Graphical Abstract

**Figure:**
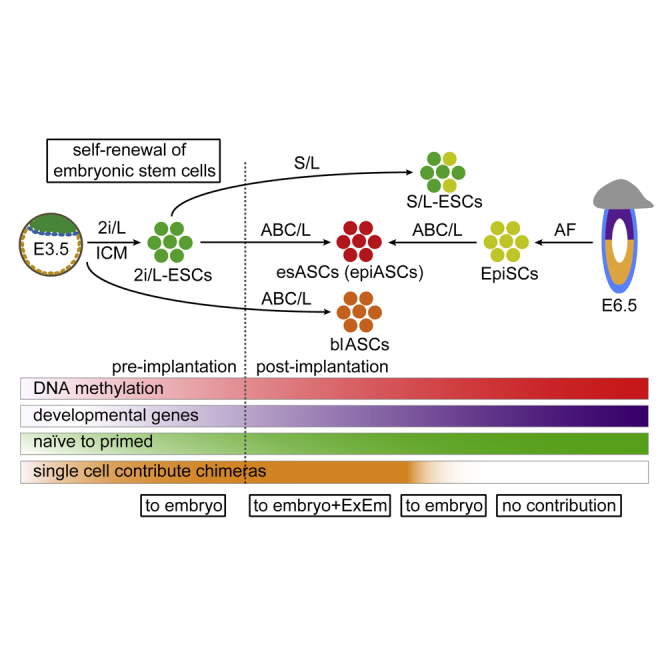

## Teaser

In this article, Bao and colleagues show that ASCs cultured in Activin A and BMP4, and in the absence of MEK1/2 inhibitor, possess reversible epigenetic changes that extend their developmental potency, distinct transcriptome pattern, and a stable hypermethylated epigenome. Importantly, they also derived novel hypermethylated ASCs from blastocysts in ABC/L medium.

## Introduction

Mouse embryonic stem cells (ESCs) were originally derived by coculture with a feeder layer of mitotically inactivated fibroblasts in medium containing fetal calf serum (Evans and Kaufman, 1981, Martin, 1981). It was later shown that feeder cells could be replaced by the cytokine leukemia inhibitory factor (LIF) (Smith et al., 1988), and that serum could be substituted by bone morphogenetic protein (BMP) (Ying et al., 2003). However, ESCs cultured in BMP plus LIF medium show low single-cell clonogenic capacity, and are difficult to maintain in a homogeneous state (Ying et al., 2003). Inhibitors of Mek1/2 (PD0325901, PD) and Gsk3β (CHIR99021, CH), known as 2i, enhanced the derivation of ESCs (hereafter termed 2i/L-ECSs) and promoted ground-state pluripotency (Ying et al., 2008), also known as “naive pluripotency” (Nichols and Smith, 2009), when LIF was added. Recent reports show that prolonged culture with PD impairs the developmental potential and genomic stability of mouse ESCs. Defects that are efficiently rescued by serum (Choi et al., 2017, Yagi et al., 2017) return mouse ESCs to their original culture condition and original state. Thus, how cell stability, self-renewal, and pluripotency are specifically maintained in ESCs still remain largely unknown.

We recently developed a culture system without serum and feeder, to derive advanced embryonic stem cells (ASCs), and demonstrated that such pluripotent stem cells possess enhanced *in vivo* developmental potential and unique self-renewing features when compared with mouse 2i/L-ESCs (Bao et al., 2018). We thus hypothesized that a similar experimental paradigm of targeting key developmental pathways could replace serum and feeder, applied for establishing stable hypermethylated embryonic stem cells from control ESCs and EpiSCs (Brons et al., 2007, Tesar et al., 2007). Here, we targeted four components of different signaling pathways as follows: Activin A (Act A), BMP4, and two components of 2i/L, CH, and LIF. Act A and BMP4 belong to the transforming growth factor β family of ligands. Act A promotes activation of the SMAD2/3 transcription factors, considered beneficial for self-renewal of human ESCs (Vallier et al., 2005) and mouse EpiSCs derivation (Brons et al., 2007, Tesar et al., 2007). BMP4 inhibits differentiation genes and sustains self-renewal of mouse ESCs in collaboration with STAT3 (Williams et al., 1988, Ying et al., 2003). CH acts via inhibition of GSK3 to enhance mouse ESC growth and LIF drives STAT3-dependent self-renewal (Ye et al., 2016, Ying et al., 2008). Counterintuitively we replaced PD in 2i/L-ESC medium with Act A and BMP4, whose actions directly oppose those of PD and function to promote the development of post-implantation embryo and lineage specification. Hereafter, we refer to the medium containing this cocktail of different signaling pathways as ABC/L medium, and used it for derivation of stable stem cell lines from 2i/L-ESCs, EpiSCs, and directly from blastocysts. Using ABC/L medium we have replaced the original coculture system of feeder and serum, and established ESCs with higher developmental potential compared with 2i/L-ESCs.

## Results

### ABC/L Converts ESCs into esASCs with High Genomic Stability

**Figure 1:**
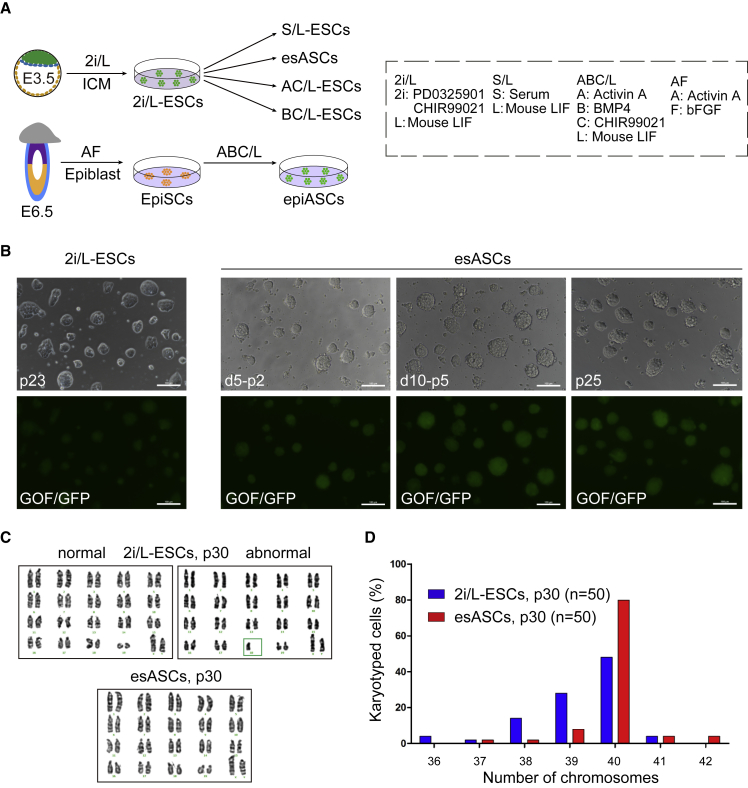
Characteristics of esASCs and epiASCs (A) Experimental outline of the esASCs derivation procedures from ESCs and EpiSCs. (B) 2i/L-ESCs (p23) were switched to ABC/L medium and cultured for 5, 10 days (d5, d10) and passages 25 (esASCs, p25). Here, we use 2i/L-ESCs with GOF/GFP reporter. Scale bars, 100 μm. (C) Karyotyping of 2i/L-ESCs (P30) and esASCs (P30). (D) Distribution of chromosome number in 2i/L-ESCs (p30) and esASCs (p30). n (n = 50), number of spread analyzed and obtained from 2 independent experiments.

We previously reported that ABC/L medium converted blastocyst-derived AFSCs into ASCs (Bao et al., 2018). In this study, we investigated if ABC/L can also convert mouse 2i/L-ESCs with *Oct4* distal enhancer (GOF/GFP) (Yoshimizu et al., 1999) (Figure 1A). The 2i/L-ESCs that were derived from blastocysts in the presence of 2i and LIF medium with N2B27 (basic medium used in this study, which excludes serum, knockout serum replacement, and feeder cells), survived well in ABC/L medium and proliferated similarly to 2i/L-ESCs (henceforth called esASCs); importantly these cells self-renewed for more than 30 passages (Figures 1B and S1A). We tested five different 2i/L-ESC cell lines (E14, SQ3.3, SQ3.4, WG3-1, and WG3-2), and all five lines were converted to esASCs.

We also investigated whether ABC/L medium was able to convert EpiSCs to stable ESCs (Figure 1A). EpiSCs are derived from early post-implantation embryos and are distinct from ESCs in culture properties, gene expression, pluripotency, and epigenetic profiles (Brons et al., 2007, Tesar et al., 2007). Some molecular features of EpiSCs are similar to AFSCs; however, they are derived from different embryonic stages. Using ABC/L, we successfully converted EpiSCs to stable ESCs (Figures S1B and S1C). EpiSCs converted to ESCs (hereafter termed epiASCs) while maintaining pluripotent properties similar to 2i/L-ESCs with GOF/GFP reporter (Figures S1B and S1C–S1E), two active X chromosomes in female epiASCs (Figures S1F–S1H), teratoma formation with multiple tissue types (Figure S1I), and possess normal karyotype (Figures 1C, 1D, and S1J). A previous report showed that it is difficult to convert EpiSCs to ESCs in 2i/LIF medium (Hall et al., 2009). Here, our data suggested that Act A and BMP4 replacing PD improves the genomic stability of ESCs.

### esASCs Display Distinct Molecular Features for Pluripotency and Developmental Stage

**Figure 2:**
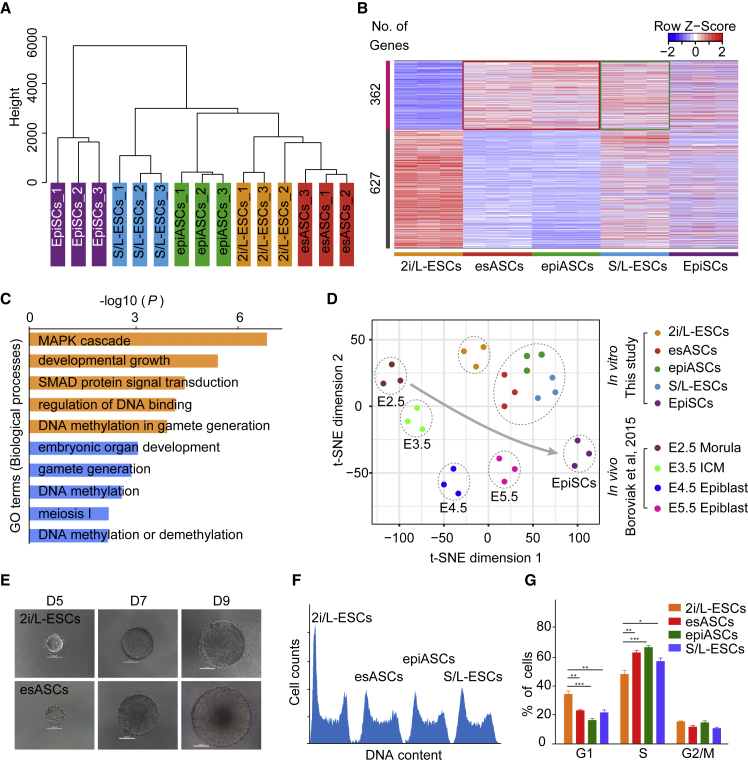
Analyses of Molecular Features of esASCs and epiASCs (A) Unsupervised hierarchical clustering (UHC) of the transcriptome from three biological replicates of five stem cell lines. Note that esASCs were clustered close to 2i/L-ESCs. (B) Heatmap showing scaled expression values of a total of 989 differentially expressed genes (mean log2(normalized read counts) > 2, log2(fold change) > 2, adjusted p value < 0.05) in esASCs and epiASCs compared with 2i/L-ESCs, S/L-ESCs, and EpiSCs. (C) The top representative GO terms (biological process) for esASC- and epiASC-upregulated genes. (D) t-SNE analysis of gene expression of pluripotent stem cells, and of E2.5–E5.5 embryos, based on 1,685 dynamically expressed genes. Arrow indicates developmental progression from E2.5 morula to E5.5 post-implantation epiblast. (E) The colonies derived by single cells of 2i/L-ESCs and esASCs. Scale bars, 100 μm. (F) Representative image of FACS-based cell-cycle analysis on 2i/L-ESCs, esASCs, epiASCs, and S/L-ESCs. (G) G1, S, and G2/M phase were compared to four stem cell lines. Error bars indicate SEM (n = 3). Results were obtained from three independent experiments. Significance was tested using the two-tailed unpaired Student's t test, p < 0.05.

To examine whether esASCs have distinct molecular features (Trapnell et al., 2009) and correspond to a particular embryonic developmental stage (Boroviak et al., 2015), we compared esASCs (passage 19, p19) and epiASCs (p11) global expression dynamics with 2i/L-ESCs (p21), S/L-ESCs (2i/L-ESC culture in serum + LIF medium, p12), and EpiSCs (p15). Unsupervised hierarchical clustering (UHC) shows esASCs close to 2i/L-ESCs (Figure 2A) and a total of 989 differentially expressed genes (DEGs) (Robinson et al., 2010) in esASCs and epiASCs compared with 2i/L-ESCs (Figure 2B). A total of 362 upregulated genes in esASCs and epiASCs are also upregulated in S/L-ESCs (Figure 2B). Gene ontology (GO) analysis indicated that upregulated genes in esASCs were associated with the MAPK cascade, developmental growth, and regulation of DNA binding (Figure 2C). To benchmark developmental progression, we compared our esASCs with *in vivo* embryonic day (E) E2.5–E6.5 embryos (Boroviak et al., 2015). t-SNE analysis showed that esASCs were intermediate between E4.5 and E6.5 and close to S/L-ESCs (Figures 2D and S2A).

Interestingly, among DEGs (Ernst and Bar-Joseph, 2006), 1,834 genes were related to stem cell development, pattern specification process, and mesoderm development, these genes gradually increased from 2i/L-ESCs, esASCs, epiASCs, S/L-ESCs, and EpiSCs (Figures S2B and S2C). Notably, a total of 1,303 genes were significantly upregulated in esASCs and epiASCs compared with 2i/L-ESCs, S/L-ESCs, and EpiSCs (Figure S2B). GO analysis indicated that upregulated genes in esASCs and epiASCs were associated with methylation, covalent chromatin modification, and DNA replication, including synthesis of DNA, G1/S transition, and cell-cycle checkpoint processes (Figure S2C). We also found that the expression of pluripotency core factors do not change (except for *Prdm14* and *Nanog*) between 2i/L-ESCs and esASCs, whereas the transcriptional level of genes known to influence DNA methylation levels, such as *Prdm14* and *Nanog*, were significantly downregulated in esASCs compared with 2i/L-ESCs (Figure S2D). Transcription factors, genes regulating signal pathways, and epigenetic modification-associated genes that are upregulated in the post-implantation epiblast (Chen et al., 2018, Kalkan et al., 2017, Tsanov et al., 2017) were similarly induced in esASCs and epiASCs (Figure S2E). These RNA sequencing (RNA-seq) analyses suggest that esASCs and epiASCs are in a stable state between naive and primed pluripotency and more similar to S/L-ESCs.

### esASCs Exhibit Intermediate Features between 2i/L-ESCs and EpiSCs with a Distinct Self-Renewal Property

To understand the properties and propagation efficiency of esASCs in ABC/L medium, we examined the single-cell clonogenicity and cell progression of esASCs. Single esASCs and 2i/L-ESCs were transferred to 96-well dishes in N2B27 with addition of ABC/L and 2i/L, respectively. esASCs exhibit similar single-cell clonogenicity to 2i/L-ESCs (Figures 2E and S2F), but possess larger clone diameter (Figures 2F and S2G) and show increased cellular proliferation (Figure S2H). The results of fluorescence-activated cell sorting (FACS)-based cell-cycle analysis shows that more esASCs and epiASCs reside in the S phase compared with 2i/L-ESCs, which showed more cells in the G1 phase (Figures 2F and 2G). This result suggests that the cell-cycle control in esASCs and epiASCs is similar to that in S/L-ESCs, consistent with our RNA-seq analysis, which revealed that cell-cycle-associated genes in esASCs were close to S/L-ESCs and distinct from 2i/L-ESCs (Figures S2C and S2I). Indeed, we found that the level of c-MYC in esASCs was significantly higher than 2i/L-ESCs (Figure S2J), and that the target of Wnt/β-catenin pathway protein CDX1 (Pilon et al., 2007) was strongly expressed in our esASCs (Figure S2K). Together, these results suggest that esASCs have a distinct self-renewing type of pluripotent stem cells and exhibit intermediate features between 2i/L-ESCs and EpiSCs.

### Act A and BMP4 Improves the Developmental Potency of ESCs

**Figure 3:**
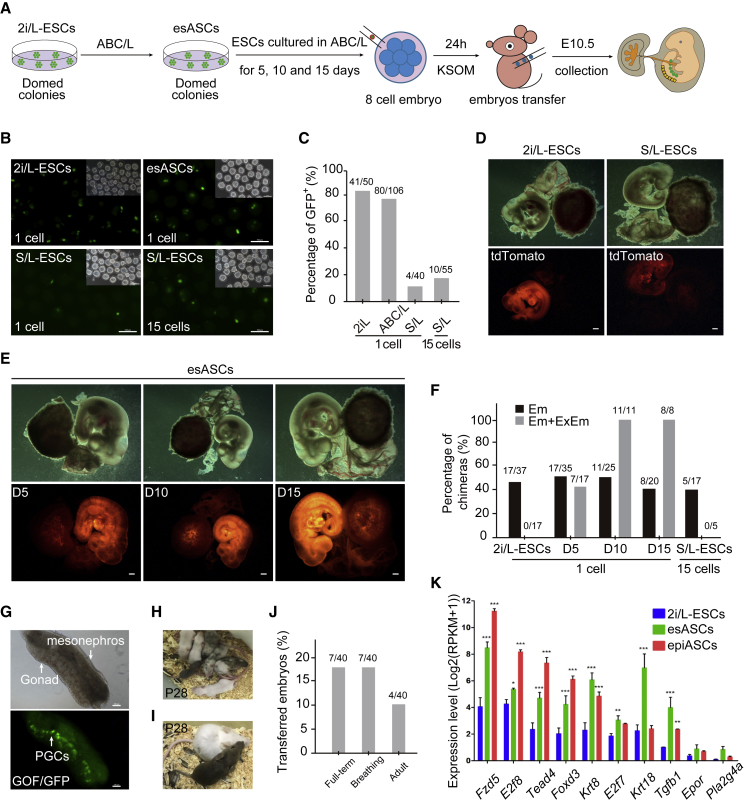
Act A and BMP4 Expand the Developmental Potency of ESCs (A) Schematic of eight-cell embryo injection protocol. (B) Representative image of three kinds of ESCs (GOF/GFP) were introduced into eight-cell embryos and cultured for 24 h. Scale bars, 100 μm. Results were obtained from two independent experiments. (C) Percentage of GOP/GFP positive embryos after 24 h *in vitro* culture, at three kinds of ESCs. Results were obtained from two independent experiments. (D) Chimeras (E10.5) generated with 2i/L-ESCs (single cell) and S/L-ESCs (multiple cells). ESCs carried tdTomato^+^/GOF^+^ reporter. Scale bars, 1 mm. (E) Single esASCs contributed to embryo, yolk sac, and placental labyrinth in E10.5 chimeras. Scale bars, 1 mm. (F) Summary of E10.5 chimera assays by ESC injection. Single cell of 2i/L-ESCs, 2i/L-ESCs cultured in ABC/L medium for 5, 10, and 15 days, respectively, and 15 cells of S/L-ESCs were injected into eight-cell stage embryos, and transferred to pseudopregnant mice, and the chimeras were collected at E10.5. The black bar chart shows the percentages of chimeras among the collected E10.5 conceptus embryonic tissues (Em); gray bar, integration into both embryonic and extraembryonic mesoderm (Em + ExEm) among the recovered E10.5 chimeras. Results were obtained from two independent experiments. (G) Germline transmission of esASCs in E12.5 chimeras. PGCs were shown by GOF/GFP^+^ cells (arrow). Scale bars, 100 μm. (H) Chimeric pups generated by injecting single esASCs in ICR host blastocysts. Detailed data are provided in Figure S3G. (I) Pups generated entirely from esASCs in tetraploid ICR host blastocysts by injecting about 15 cells. (J) Statistics of full-term ASCs derived mice. Results were obtained from two independent experiments. (K) Expression levels (log2(RPKM + 1)) of placenta development-related genes in 2i/L-ESCs, esASCs, and epiASCs. Error bars indicate SEM (n = 3). Results were obtained from three independent experiments. p values were calculated by two-way ANOVA, p < 0.05.

The functional attribution of mouse ESCs is their capacity to re-enter embryonic development and contribute to differentiated tissues in chimeric mice. We proceeded to demonstrate the *in vivo* developmental potency of esASCs by injecting H2B tdTomato reporter and GOF/GFP-positive (tdTomato^+^/GOF^+^) single esASCs, epiASCs, S/L-ESCs, and control 2i/L-ESCs into eight-cell embryos and investigated chimeric development (Figure 3A). First, we assessed single GOF/GFP^+^ stem cells maintaining developmental rate at 24 h after injection, and found that GOF/GFP^+^ cells (4/40) in the S/L-ESCs group had significantly lower rates than esASCs and 2i/L-ESCs (Figures 3B and 3C). Second, we analyzed their contribution to chimeric embryos at E10.5 and found that tdTomato-positive esASC and epiASC donor cells contributed robustly to the embryo proper, yolk sac, and placental labyrinth (Figures 3E and S3A–S3C). Whereas 2i/L-ESCs contributed to the embryo, and slightly contributed to the yolk sac, as well as placental labyrinth (Figure 3D), no chimeric embryos were found in S/L-ESC groups upon single-cell injection. When we injected 15–20 cells of S/L-ESCs into eight-cell embryos, chimeric embryos collected contributed similarly to 2i/L-ESCs in E10.5 (Figures 3B–3D). Notably, the high contribution of esASCs to yolk sac was only in the extraembryonic mesoderm, and there was no detectable contribution to the extraembryonic endoderm of the yolk sac (Figure S3C). These observations suggest that esASCs and epiASCs are stable and possess a higher potency to contribute to embryos and placentas compared with S/L-ESCs and 2i/L-ESCs. Furthermore, we show that S/L-ESCs are highly heterogeneous and that single-cell injection fails to contribute to embryo in chimeras.

To further assess the robustness of Act A and BMP4 in the medium, we switched 2i/L-ESCs (p20) back to ABC/L medium for an additional 5, 10, and 15 days culture and detected their single-cell contribution ability to chimeric embryos at E10.5 (Figure 3A). We found that only 5 days of ABC/L culture resulted in an increased contribution to yolk sac and placental labyrinth, and the rate was further increased after 10 and 15 days of ABC/L culture (Figures 3E and 3F). These results confirm that the function of Act A and BMP4 is to expand the pluripotency of 2i/L-ESCs completed in ABC/L medium for 10 days. Furthermore, esASCs cultured from 2i/L-ESCs were able to contribute to germline lineages (Figures 3G, S3D, and S3E), full-term chimeras (Figures 3H, S3F, and S3G), and tetraploid complementation (Figures 3I and 3J). However, the ratio of esASC-derived teraploid pups (10%, 4/40) was similar to S/L-ESCs in the multiple-cell injection (Choi et al., 2017). This result further suggests that esASCs possess stable cell-renewing ability with a strong potential to contribute to embryos, yolk sac, and placental labyrinth. Interestingly, RNA-seq data suggested that placenta development-related genes were significantly upregulated in esASCs when compared with 2i/L-ESCs (Figure 3K). Particularly, knockout of *Fzd5*, *E2f7/8*, and *Krt8* in ESCs critical affects the development of placenta (Ishikawa et al., 2001, Jaquemar et al., 2003, Li et al., 2008, Ouseph et al., 2012). These transcriptional differences in placenta genes partially explain the mechanism of the enhanced potency of esASCs in chimera formation.

### Activin A or BMP4 Could Replace Mek1/2 Inhibitor to Maintain the Developmental Potency of ESCs

To explain which signaling pathway plays a critical role in the ABC/L culture system, we prevented different signaling by using appropriate inhibitors, and selected epiASCs to perform the experiments. We first removed Act A, BMP4, CHIR99021, and LIF, respectively, from ABCL medium, and accordingly added inhibitors of SB431542, Noggin, XAV939, and JAK inhibitor I to prevent Activin A, BMP4, Wnt, and JAK/STAT3 signaling. The five groups of media (ABCL medium, A^−^ [BCL + SB431542], B^−^ [ACL + Noggin], C^−^ [ABL + XAV939], and L^−^ [ABC + JAK inhibitor I]) were cultured with epiASCs for 6 days. We detected that Wnt (C^−^) and JAK/STAT3 (L^−^) signaling was critical to the self-renewal of epiASCs, qPCR results in C^−^ and L^−^ show key pluripotent genes decreased and strong expression of some epiblast and mesoderm genes in 6-day cultures (Figures S4A and S4B). Together, these data suggest that Wnt and JAK/STAT3 signaling pathways were largely responsible for regulating the self-renewal of ASCs.

**Figure 4:**
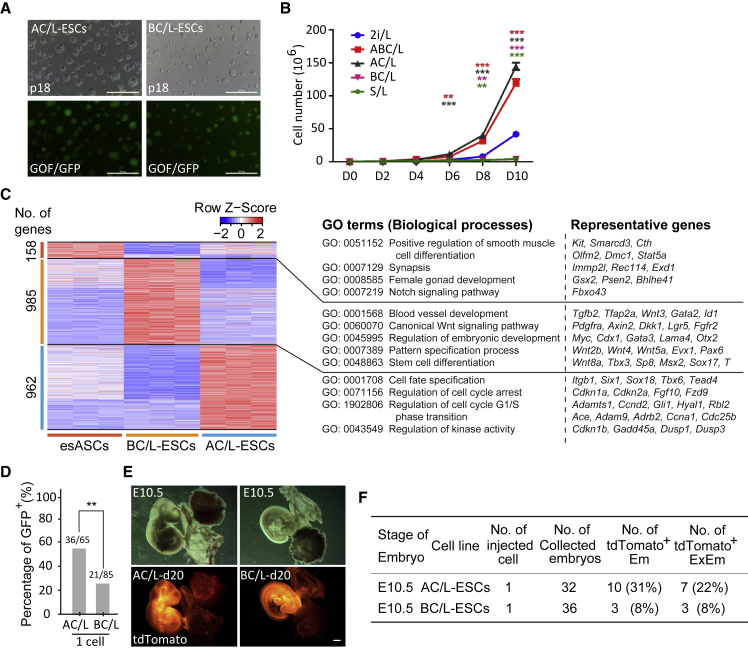
The Role of Act A or BMP4 in Maintaining esASCs Developmental Potency (A) Morphology of 2i/L-ESCs were switched to AC/L or BC/L medium at passage 18 (p18). Here, we use 2i/L-ESCs with GOF/GFP reporter. Scale bars, 200 μm. (B) Cell proliferation curves in ESCs cultured in five different mediums. Error bars indicate SEM (n = 3). Results were obtained from three independent experiments. p values were calculated by two-way ANOVA, p < 0.05. (C) Heatmap showing scaled expression values of a total of 2,105 DEGs (mean log2(normalized read counts) > 2, log2(fold change) > 2, adjusted p value < 0.05) in AC/L-ESCs, BC/L-ESCs, and esASCs. Significantly enriched GO terms and representative genes in each cluster are listed on the right. (D) Single AC/L-ESCs or BC/L-ESCs (GOF/GFP) were introduced into eight-cell embryos and the GOF/GFP^+^ embryos were counted after 24-h *in vitro* culture. Error bars indicate SEM (n = 3). Results were obtained from three independent experiments. Significance was tested using the two-tailed unpaired Student's t test, p < 0.05. (E) Chimeras (E10.5) generated with AC/L-ESCs (single cell) or BC/L-ESCs (single cell). The images show that ESCs carried tdTomato^+^/GOF^+^ reporter. Scale bars, 1 mm. (F) Summary of E10.5 chimera assays by ESCs injection.

Next, to explain whether signaling of Activin A or BMP4 plays a more important role in the ABC/L culture system, we added Activin A or BMP4 with CHIR99021 and LIF, named AC/L and BC/L culture media, respectively. When 2i/L-ESCs (GOF/GFP reporter) were switched to AC/L (AC/L-ESCs) or BC/L (BC/L-ESCs) medium, the morphologies were similar to ESCs cultured in 2i/L medium (Figure 4A). AC/L-ESCs and esASCs possess increased cellular proliferation compared with 2i/L-ESCs (Figure 4B). However, the cellular proliferation of BC/L-ESCs is significantly slower than that of 2i/L-ESCs (Figure 4B).

In addition, the gene expression pattern showed that there were a total of 962 upregulated genes in AC/L-ESCs, and a total of 985 upregulated genes in BC/L-ESCs, compared with esASCs (Figure 4C). GO analysis indicated that upregulated genes in AC/L-ESCs were associated with the regulation of cell cycle, cell fate specification, and regulation of kinase activity (Figure 4C), whereas the upregulated genes in BC/L-ESCs were associated with blood vessel development, regulation of embryonic development, pattern specification process, and stem cell differentiation (Figure 4C).

To understand which signaling pathway contributes to the developmental potency of AC/L-ESCs and BC/L-ESCs, we introduced single cells (AC/L-ESCs or BC/L-ESCs) into eight-cell stage mouse embryos. After 24 h *in vitro* culture, we tested single GOF/GFP^+^ stem cells maintaining developmental rate, and found more GOF/GFP^+^ cells (36/65) in the AC/L-ESCs, significantly higher than BC/L-ESCs (21/85) (Figure 4D). Our results also showed that ESCs cultured in either AC/L or BC/L contributed to the placenta and the yolk sac in chimeras; however, the efficiency of the AC/L-ESCs is higher than the BC/L-ESCs (Figures 4E and 4F).

### Global Upregulation of DNA Methylation Level in esASCs and epiASCs

**Figure 5:**
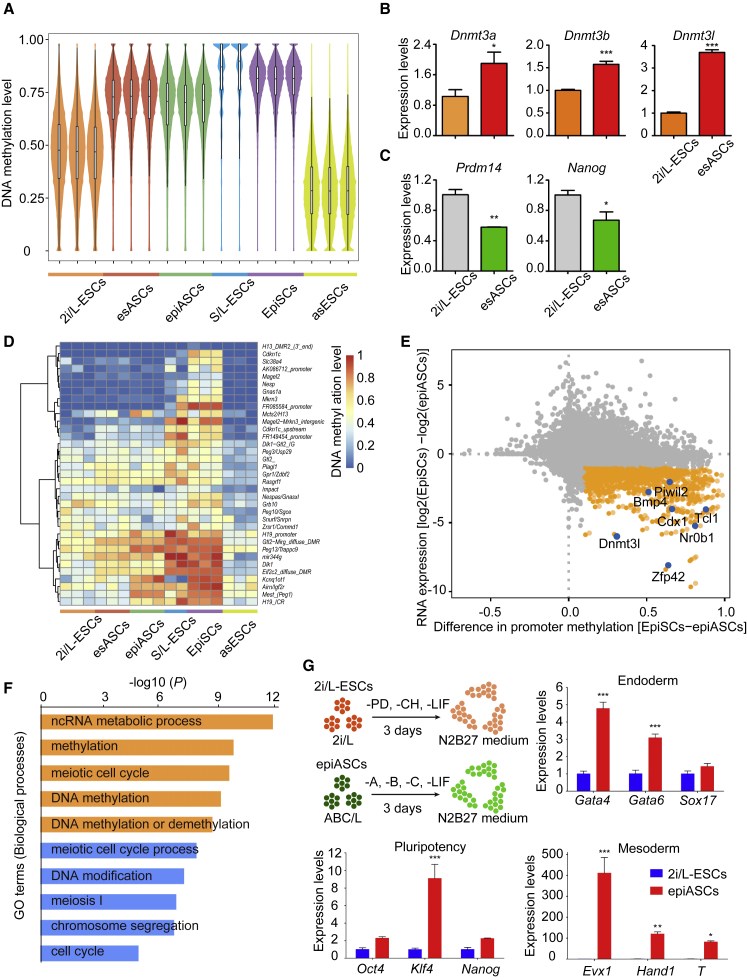
Global Upregulation of DNA Methylation Level in esASCs and epiASCs (A) Violin plot showing DNA methylation level of 2-kb genomic tiles. asESCs indicate that ASCs were cultured in 2i/L medium. Source data are provided in Table S1. (B) Relative expression of DNA methyltransferase genes (*Dnmt3a, Dnmt3b* and *Dnmt3l*) measured by qPCR in esASCs. 2i/L-ESCs were used as control. Error bars indicate SEM (n = 3). Results were obtained from three independent experiments. p values were calculated by unpaired Student's t test, p < 0.05. (C) Relative expression of DNA methylation-associated genes (*Prdm14* and *Nanog*) measured by qPCR in esASCs. 2i/L-ESCs were used as control. Error bars indicate SEM (n = 3). Results were obtained from three independent experiments. p values were calculated by unpaired Student's t test, p < 0.05. (D) Heatmap showing DNA methylation level of ICRs in five different stem cells. (E) Scatterplot of differential gene expression and difference in promoter DNA methylation level between EpiSCs and epiASCs. Genes upregulated in epiASCs with promoter demethylation are highlighted in orange. Source data are provided in Table S2. (F) The top representative GO terms for Figure 5E upregulated and promoter demethylation genes. (G) Experimental outline of ESCs differentiation. Mesoderm, endoderm, and pluripotency-associated genes were tested by qPCR, epiASCs were checked after 3 days induction, 2i/L-ESCs were used as control. Error bars indicate SEM (n = 3). Results were obtained from three independent experiments. p values were calculated by two-way ANOVA, p < 0.05.

It is known that S/L-ESCs have higher DNA methylation levels compared with 2i/L-ESCs (Ficz et al., 2013, Habibi et al., 2013, Hackett et al., 2013, Yagi et al., 2017). Here, we analyzed DNA methylation profiles of 2i/L-ESCs (p13), esASCs (p12), epiASCs (p13), and EpiSCs (p13) by whole-genome bisulfite sequencing (WGBS) (Krueger and Andrews, 2011) (Figure 5A). We found that global DNA methylation levels of esASCs (median CpG methylation level 74.6%) and epiASCs (median 72.2%) were higher than 2i/L-ESCs (median 48.1%) and similar to EpiSCs (median 83.3%) and S/L-ESCs (median 88.9%) (Figure 5A) (Choi et al., 2017, Hackett et al., 2017). This methylation pattern occurs across most genomic regions (Figure S5A), including exons, introns, intergenic and intragenic regions, promoters, and nuclear repeat elements (SINEs, LINEs, LTRs, and Satellite, respectively). To understand how ABC/L medium induces global DNA hypermethylation, we checked the mRNA and protein levels of methyltransferases in 2i/L-ESCs and esASCs. As expected, DNA methyltransferases (*Dnmt1*, *Dnmt3a*, *Dnmt3b*, and associated cofactor *Dnmt3l*) were upregulated at transcriptional and protein levels in esASCs (Figures 5B and S5B). Transcriptional levels of genes known to influence DNA methylation levels, such as *Prdm14* and *Nanog*, were significantly downregulated in esASCs (Figure 5C). Notably, regulators of MAPK-ERK signaling were upregulated in esASCs (Figure S5C). In addition, DNA methylation at imprinting control regions (ICRs) (Xie et al., 2012) were erased in 2i/L-ESCs, whereas most DNA methylation at ICRs was retained in esASCs, epiASCs, S/L-ESCs, and EpiSCs (Figures 5D and S5D). Analysis of the difference in promoter methylation and gene expression between epiASCs and EpiSCs showed that, compared with EpiSCs, a small fraction of genes (796) were upregulated in epiASCs and exhibited promoter demethylation (Figure 5E). These genes were involved in methylation, meiotic cell cycle, and DNA modification (Figure 5F). Importantly, we converted ASCs back to 2i/L culture condition (referred to as asESCs) and performed WGBS, the data showed that the DNA methylation levels of asESCs were convertible (Figures 5A, 5D, and S5A). We conclude that ABC/L culture causes a reversible effect on global DNA methylation levels and genomic imprints upon 2i/L-ESCs.

In addition, upon 2i and LIF withdrawal, pluripotent ESCs differentiate into three germ layers, mesoderm, endoderm, and ectoderm (Shen et al., 2008). To investigate the differentiation capacity of epiASCs, we withdrew four factors (Act A, BMP4, CH, and LIF) over 3 days (Figure 5G) and performed qPCR analysis. Compared with withdrawal of 2i and LIF from 2i/L-ESCs, withdrawal of the four factors from epiASCs significantly increased mesoderm and endoderm markers (Figure 5G). The expression of pluripotent genes decreased in general, but the expression level of *Oct4*, *Nanog*, and *Klf4* were relatively high in epiASCs after 3 days of culturing compared with 2i/L-ESCs (Figures 5G, S5E, and S5F). These results show that esASCs derived from 2i/L-ESCs, exist in a condition of “precarious balance” (Loh and Lim, 2011), but are simultaneously allowed to poise for multi-lineage induction, due to their higher differentiation ability and unique pluripotent state compared with 2i/L-ESCs.

### ABC/L Is Capable of Establishing Intermediate ESCs Directly from Blastocyst

**Figure 6:**
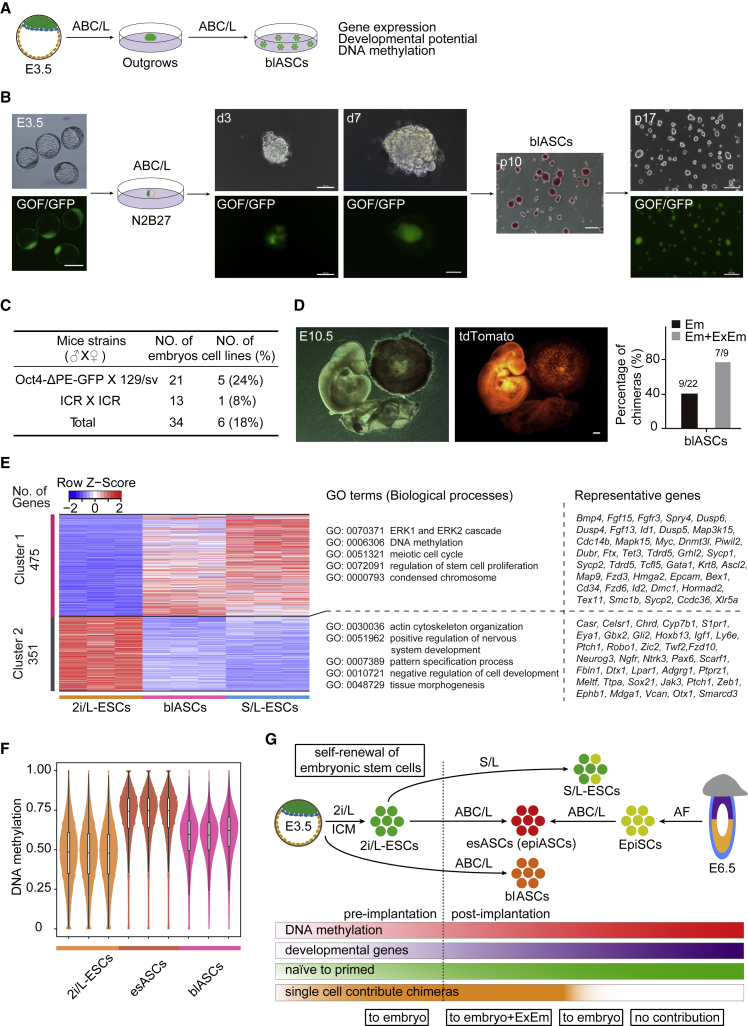
Characteristics of blASCs Directly Derived from Blastocysts (A) Experimental outline of blASCs derived from blastocysts. (B) GOF/GFP and AP-positive blASCs lines were directly derived from blastocysts. The blASCs stable passages over 20. Scale bar, 100 μm. (C) Derivation rate of blASCs from blastocysts. (D) The blASCs contributed to embryo, yolk sac, and placental labyrinth in E10.5 chimeras. Scale bars, 1 mm. Summary of E10.5 chimera assays (right) by single blASCs injection. The black bar chart shows the percentage of chimeras among the collected E10.5 conceptus embryonic tissues (Em); gray bar, integration into both embryonic and extraembryonic mesoderm (Em + ExEm) among the recovered E10.5 chimeras. Results were obtained from two independent experiments. (E) Heatmap showing scaled expression values of a total of 826 DEGs (mean log2(normalized read counts) > 2, log2(fold change) > 2, adjusted p value < 0.05) in blASCs and S/L-ESCs compared with 2i/L-ESCs. Significantly enriched GO terms and representative genes in each cluster are listed on the right. (F) Violin plot showing DNA methylation level of 2-kb genomic tiles. Source data are provided in Table S1. (G) Schematic representation of self-renewal of different ESC lines in mice.

Finally, as a stringent test of robustness of ABC/L medium, we attempted to derive novel ESCs directly from blastocysts (Figure 6A). As expected, we established GOF/GFP^+^ advanced pluripotent stem cells derived from blastocysts (referred to as blASCs) under ABC/L conditions from 129/sv and ICR background mice (Figures 6B and 6C). Blastocysts were cultured into ABC/L medium, the GOF/GFP^+^ inner cell mass (ICM) cells were retained well and proliferated to around 200 μm in diameter after 7 days of culture. This colony was cut into smaller pieces using glass needles and then transferred into new culture plate. When these colonies had grown for 6–7 days, they were treated with Accutase, and the resulting cells were cultured to produce GOF/GFP^+^ colonies, which were capable of self-renewal for over 20 passages and AP positive (Figure 6B). Furthermore, blASCs exhibited the same pluripotency, such as expression of Oct4, Sox2, and Nanog; c-MYC and CDX1 were also significantly expressed in blASCs (Figures S6A and S6B). We further demonstrated that the *in vivo* developmental ability of blASCs was similar to esASCs, single blASCs donor cells contributed robustly to the embryo proper, yolk sac, and placental labyrinth (Figure 6D). Notably, the gene expression patterns of blASCs were also similar to S/L-ESCs (Figures 6E, S6C, and S6D), a total of 475 upregulated genes in blASCs are also upregulated in S/L-ESCs (Figure 6E). GO analysis indicated that upregulated genes in blASCs and S/L-ESCs were associated with the ERK cascade, DNA methylation, meiotic cell cycle, regulation of stem cell proliferation, and condensed chromosomes (Figure 6E). Importantly, blASCs exhibit normal karyotyping (Figure S6E), high global DNA methylation level (Figure 6F), and most imprints were retained (Figure S6F). This result suggested that ABC/L medium could establish intermediate ESCs directly from blastocysts and support long-term *in vitro* culture. Furthermore, single blASC donor cells contributed robustly to the embryo proper. Therefore, our data show that blASCs are a novel type of stem cells which is an intermediate state between the naive and primed states.

## Discussion

Despite the great application potential of ESCs, the quality and stability of ESCs still remains to be improved; however, the mechanism underlying genetic and epigenetic modification following different culture media remains largely undefined. Here, we show that a chemically defined medium enables the conversion of 2i/L-ESCs to esASCs, as well as derivation of blASCs directly from blastocysts (Figure 6G). We demonstrate that esASCs converted from 2i/L-ESCs contributed to germline lineages and full-term chimeras, and generated esASC-derived mice by tetraploid complementation. We show that esASCs possess distinct molecular features, a stable DNA hypermethylated epigenome, and higher genomic stability (Figure 6G). Furthermore, the cell-cycle control of esASCs and epiASCs is different from 2i/L-ESCs but similar to S/L-ESCs, which show distinct cell-cycle control different from 2i/L-ESCs and S/L-ESCs (Ter Huurne et al., 2017). Interestingly, compared with esASCs and 2iL-ESCs, blASCs showed intermediate levels of DNA methylation (Figure 6F). The difference of methylation level is mainly due to the different origins of esASCs and blASCs. esASCs are derived from 2i/L-ESCs, and blASCs are directly established from the ICM of blastocysts. However, the precise regulatory mechanism needs to be further explored. We found that the levels of c-MYC in esASCs were significantly higher than in 2i/L-ESCs, which may associate with the cell-cycle properties of esASCs. A report showed that *c-Myc* potentiates the Wnt/β-catenin signaling pathway in ESCs (Fagnocchi et al., 2016, Pilon et al., 2007). Together, these results support the role of *c-Myc* via the Wnt/β-catenin pathway to promote self-renewal in esASCs; however, further studies are required to investigate the precise molecular mechanism. Furthermore, when we cultured 2i/L-ESCs in AC/L, cellular proliferation and chimeric effect were more similar to esASCs than BC/L-ESCs. Our results reveal that the degree of Activin A signaling dictates the epigenetic state and chromosomal stability of 2i/L-ESCs, which lies between the naive ICM and the developmentally more advanced post-implantation epiblast. In addition, CHIR99021 and LIF (CL) combination medium was sufficient to maintain the cell-renewal of ASCs (epiASCs) for at least 10 passages (data not shown).

Multiple global DNA methylome analyses have shown that S/L-ESCs are globally hypermethylated, whereas ICM cells from preimplantation embryos are hypomethylated (Habibi et al., 2013, Hackett et al., 2017, Stadler et al., 2011). The DNA methylation levels of esASCs and epiASCs were shown to be between ESCs and EpiSCs, similar to S/L-ESCs. The transcriptional levels of *Prdm14* and *Nanog* were significantly downregulated, and regulators of MAPK-ERK signaling were upregulated in esASCs compared with 2i/L-ESCs. These observations are consistent with the previous notion that *Prdm14* co-occupies with *Nanog*, and represses expression of *de novo* DNA methyltransferases (Leitch et al., 2013, Yamaji et al., 2013). These transcriptional differences partially explain the mechanism of the DNA methylation levels of esASCs. In addition, 2i/L-ESCs have reduced DNA methylation (Stadler et al., 2011), correlating to cellular transformation and chromosomal instability (Choi et al., 2017, Yagi et al., 2017). This study provides insight into the DNA methylation dynamics of ESCs, which exist in a different milieu of cell culture conditions. Also, we established ESCs directly from blastocysts using the ABC/L medium, and these results reinforce the robustness of the ABC/L medium, providing evidence that Act A and BMP4 signaling could instruct blastocysts toward self-renewal, and capture a novel developmental state *in vitro*.

In conclusion, we demonstrate that a chemically defined medium, in particular Activin A and BMP4 signaling, enables 2i/L-ESCs to convert to esASCs with a unique DNA methylation state and a specific gene expression pattern, advanced differentiation status, and greater functional potential *in vivo*. These findings enhance our understanding about the complexity of signaling pathways in pluripotency, revealing the similarities and differences between distinct pluripotent states and expanding our knowledge on both early embryonic development and the molecular mechanisms of pluripotency regulation. We demonstrate the robustness of the ABC/L culture system to establish ESCs directly from blastocysts, and it is likely that these culture conditions could be utilized in other mammalian species to generate pluripotent stem cells to uncover their precise genetic and epigenetic regulatory mechanisms.

## Experimental Procedures

### Mice

Oct4-**Δ**PE-GFP (GOF/GFP) transgenic mice (Yoshimizu et al., 1999) were used here with a mixed background of MF1, 129/sv, and C57BL/6J strains. All animal experiments were performed following the ethical guidelines approved by Animal Research Committee of Inner Mongolia University, China.

### Derivation of Mouse ESCs

Mouse embryo blastocysts (E3.5) were isolated from 129/sv females mated with GOF/GFP transgenic males. Green fluorescence indicated that GFP expression of the reporter was under the control of the *Oct4* promoter and distal enhancer. This GFP transgene shows expression in the ICM of blastocysts and PGC *in vivo*, and in ESCs (Yoshimizu et al., 1999). Further information is provided in Supplemental Experimental Procedures.

### Flow Cytometry

ESCs were harvested by Accutase and fixed for 30 min in 3.7% paraformaldehyde at room temperature. After fixing, cells were incubated with Hoechst 33342 (Invitrogen) for 1 h at 37°C and analyzed on the BD LSRFortessa. Further information is provided in Supplemental Experimental Procedures.

### Western Blot

Cells were collected with trypsin, washed 3 times with PBS, and lysed in buffer that contained 20 mM Tris (pH 8.0), 137 mM NaCl, 100 g/L glycerol, 50 g/L Triton X-100, and 4 g/L EDTA; 1 μl PMSF (0.1 M) and 10 μl phosphatase inhibitor (10 g/L) were added per 1 ml lysis buffer immediately before use. Proteins were denatured with 2 × SDS at 95°C for 5 min. A total of 20 μg denatured protein was run on 8% or 10% SDS-PAGE gel and transferred to polyvinylidene difluoride membrane. Membranes were blocked with 5% nonfat milk in 1 × TBS with 0.05% Tween 20 (TBST) for 1 h. Samples were probed with primary antibodies overnight at 4°C. The primary antibodies used were anti-DNMT3A (CST, 3598S; dilution 1:1,000), anti-DNMT3B (Abcam, ab78922; dilution 1:2,000), anti-DNMT3L (Abcam, ab3493; dilution 1:2,500), anti-DNMT1 (Abcam, ab19905; dilution 1:1,000), and anti-β-ACTIN (Abcam, ab8227; dilution 1:5,000). Blots were rinsed with TBST. Membranes were incubated with horseradish peroxidase-conjugated secondary antibodies for 60 min at room temperature, and proteins were detected by ECL plus reagent. After rinsing with TBST, Clarity Western ECL Substrate (Bio-Rad) was used for visualization, and ChemiDoc MP Imaging System (Bio-Rad) was used for band detection.

### Production of Chimeras

A single cell was injected gently into the ICR mice eight-cell stage embryos using a piezo-assisted micromanipulator attached to an inverted microscope. The injected embryos were cultured in KSOM medium (Millipore) at 37°C in a 5% CO_2_ atmosphere overnight and then transferred to the uteri of pseudopregnant ICR mice at 2.5 days post coitus. The embryos were isolated at embryonic stage E9.5–E13.5 to check chimeric contribution. Full-term chimeras were confirmed by the coat color pattern of the pups at birth.

### Real-Time PCR

Total RNA was isolated with the RNeasy Plus Mini Kit (QIAGEN) and reverse transcribed into cDNA using the Reverse Transcription System (Promega) according to the manufacturer's instructions. Further information is provided in Supplemental Experimental Procedures.

### RNA Extraction and Sequencing

Total RNA were extracted from approximately one to two million cells using an RNeasy Mini Kit (QIAGEN) according to the recommendations of manufacturer and then an NEBNext Poly(A) mRNA Magnetic Isolation Module was used to isolate mRNA from total RNA. Using mRNA as input, the first- and second-strand cDNAs were synthesized using the NEBNext RNA First Strand Synthesis Module and the NEBNext Ultra II Non-Directional RNA Second Strand Synthesis Module, respectively. Final libraries were prepared using KAPA Hyper Prep Kits (eight PCR cycles) and sequenced on a HiSeq 4000 platform.

### RNA-Seq Data Analysis

Before alignment, raw data were first trimmed to remove reads with more than 10% low-quality bases and to trim adaptors. Then the clean reads were mapped to mouse reference genome (mm10) with TopHat (2.0.12) with default settings (Trapnell et al., 2009). HTSeq (0.6.1) was used for reads counting, and then RefSeq gene expression level was estimated using the RPKM method (reads per kilobase transcriptome per million reads). *In vivo* data of mouse embryos E2.5–E5.5 (Boroviak et al., 2015), *in vivo* data of mouse embryo E6.5 (GSM2588691/GSM2588692), and EpiLC (GSE99494) from a previous study were downloaded and identically processed. DEGs in different samples were determined using the edgeR package with fold change ≥ 2 and p ≤ 0.5 (Robinson et al., 2010). UHC analysis was performed by the R *hclust* function. t-SNE was carried out with the R *Rtsne* function. Heatmaps of select genes were performed using the R *heatmap.2* function. Principal component analysis was performed with the R *prcomp* function. GO analysis was performed using Metascape (http://metascape.org). Trend analysis of DEGs was performed using Short Time-series Expression Miner software (Ernst and Bar-Joseph, 2006).

### Genomic DNA Isolation and WGBS Library Preparation

Following the manufacturer's instructions, genomic DNA was extracted from stem cells using the DNeasy Blood & Tissue Kit (QIAGEN). Remaining RNA was removed by treatment with RNase A. Three replicated samples from each of these stem cells were used for library preparation to ensure repeatability of the experiment. In short, 2 μg of genomic DNA spiked with 10 ng of lambda DNA were fragmented to about 300 bp with Covaris S220. Next, end repair and A-ligation were performed to the DNA fragments. Methylated Adaptor (NEB) was then ligated to the DNA fragments. To reach >99% bisulfite conversion, the adaptor-ligated DNA was treated twice using EZ-96 DNA Methylation-Direct MagPrep (Zymo Research). The resulting single-strand DNA fragments were amplified by four PCR cycles using the KAPA HiFi HotStart Uracil + ReadyMix (2×). Finally, the libraries were sequenced on an HiSeq 4000 platform to generate 150-bp paired-end reads.

### DNA Methylation Data Analysis

WGBS reads were trimmed with Trim Galore (v.0.3.3) to remove adaptors and low-quality bases. Then we used Bismark (v.0.7.6) (Krueger and Andrews, 2011) to map the clean reads to mouse reference genome (mm10) with a paired-end and non-directional model, then the unmapped reads were realigned to the same genome with a single-end and non-directional model. PCR duplications were removed with command “samtools rmdup” (v.0.1.18). WGBS data of S/L-ESCs were downloaded from a previous study (GSE98517) (Hackett et al., 2017) and identically processed. The global DNA methylation level, estimated using a 2-kb window across the genome, and DNA methylation level in each genomic region was estimated based on 3× CpG sites (CpGs covered more than 3 times). Only regions with more than 3 CpGs covered were retained. Genomic annotation, such as exons, introns, and repeat regions were downloaded from the UCSC genome browser. Promoters were regions 1 kb upstream and 0.5 kb downstream of transcription start sites. ICRs were obtained from a previous study (Xie et al., 2012). For the low coverage of published S/L-ESCs data, DNA methylation level on ICRs were estimated based on 1× CpG sites. Locations of ICRs were converted with UCSC LiftOver from mm9 to mm10.

### Statistical Analyses

All values are depicted as mean ± SEM. Statistical parameters including statistical analysis, statistical significance, and n values are reported in the Figure legends and Supplementary Figure legends. Statistical analyses were performed using Prism Software (GraphPad Prism v.6). Significance differences were measured by an unpaired two-tailed Student's t test or two-way ANOVA. A value of p < 0.05 was considered significant.

## Author Contributions

B.W., X.L., F.T., and S.B. designed the research project, and prepared and approved the manuscript. B.W. and S.B. derived ASCs and performed *in vivo* embryo experiment. L.L., B.L., and B.W. analyses RNA-seq data and under the supervision of F.T. J.G. and L.L. prepared the RNA-seq library, the WGBS experiment, and analysed bisulfite sequencing data. B.W., Z.Y., B.Z., K.L., and C.W. analysed molecular properties under the supervision of S.B. and X.L. Y.C. and M.W. provided technical assistance. S.L. helped proofread the manuscript. M.A.S., X.L., F.T., and S.B. supervised different aspects of the paper.

## Accession Numbers

RNA-seq and WGBS data were deposited in the GEO under accession number GSE119985. All data that support the conclusions in the study are available from the authors on reasonable request.

## Supplemental Information

Document S1. Supplemental Experimental Procedures and Figures S1–S6

Table S1. WGBS Data Coverage and Conversion Efficiency, Related to Figure 5A

Table S2. Differential Expressed Genes and Difference in Promoter Methylation between EpiSCs and epiASCs, Related to Figure 5E

Table S3. qRT-PCR Primers, Related to Figures S4B and 5B

Document S2. Article plus Supplemental Information

## References

[bib1] Bao S., Tang W.W., Wu B., Kim S., Li J., Li L., Kobayashi T., Lee C., Chen Y., Wei M. (2018). Derivation of hypermethylated pluripotent embryonic stem cells with high potency. Cell Res..

[bib2] Boroviak T., Loos R., Lombard P., Okahara J., Behr R., Sasaki E., Nichols J., Smith A., Bertone P. (2015). Lineage-specific profiling delineates the emergence and progression of naive pluripotency in mammalian embryogenesis. Dev. Cell.

[bib3] Brons I.G., Smithers L.E., Trotter M.W., Rugg-Gunn P., Sun B., Chuva de Sousa Lopes S.M., Howlett S.K., Clarkson A., Ahrlund-Richter L., Pedersen R.A. (2007). Derivation of pluripotent epiblast stem cells from mammalian embryos. Nature.

[bib4] Chen A.F., Liu A.J., Krishnakumar R., Freimer J.W., DeVeale B., Blelloch R. (2018). GRHL2-dependent enhancer switching maintains a pluripotent stem cell transcriptional subnetwork after exit from naive pluripotency. Cell Stem Cell.

[bib5] Choi J., Huebner A.J., Clement K., Walsh R.M., Savol A., Lin K., Gu H., Di Stefano B., Brumbaugh J., Kim S.Y. (2017). Prolonged Mek1/2 suppression impairs the developmental potential of embryonic stem cells. Nature.

[bib6] Ernst J., Bar-Joseph Z. (2006). STEM: a tool for the analysis of short time series gene expression data. BMC Bioinformatics.

[bib7] Evans M.J., Kaufman M.H. (1981). Establishment in culture of pluripotential cells from mouse embryos. Nature.

[bib8] Fagnocchi L., Cherubini A., Hatsuda H., Fasciani A., Mazzoleni S., Poli V., Berno V., Rossi R.L., Reinbold R., Endele M. (2016). A Myc-driven self-reinforcing regulatory network maintains mouse embryonic stem cell identity. Nat. Commun..

[bib9] Ficz G., Hore T.A., Santos F., Lee H.J., Dean W., Arand J., Krueger F., Oxley D., Paul Y.L., Walter J. (2013). FGF signaling inhibition in ESCs drives rapid genome-wide demethylation to the epigenetic ground state of pluripotency. Cell Stem Cell.

[bib10] Habibi E., Brinkman A.B., Arand J., Kroeze L.I., Kerstens H.H., Matarese F., Lepikhov K., Gut M., Brun-Heath I., Hubner N.C. (2013). Whole-genome bisulfite sequencing of two distinct interconvertible DNA methylomes of mouse embryonic stem cells. Cell Stem Cell.

[bib11] Hackett J.A., Dietmann S., Murakami K., Down T.A., Leitch H.G., Surani M.A. (2013). Synergistic mechanisms of DNA demethylation during transition to ground-state pluripotency. Stem Cell Reports.

[bib12] Hackett J.A., Kobayashi T., Dietmann S., Surani M.A. (2017). Activation of lineage regulators and transposable elements across a pluripotent spectrum. Stem Cell Reports.

[bib13] Hall J., Guo G., Wray J., Eyres I., Nichols J., Grotewold L., Morfopoulou S., Humphreys P., Mansfield W., Walker R. (2009). Oct4 and LIF/Stat3 additively induce Kruppel factors to sustain embryonic stem cell self-renewal. Cell Stem Cell.

[bib14] Ishikawa T., Tamai Y., Zorn A.M., Yoshida H., Seldin M.F., Nishikawa S., Taketo M.M. (2001). Mouse Wnt receptor gene Fzd5 is essential for yolk sac and placental angiogenesis. Development.

[bib15] Jaquemar D., Kupriyanov S., Wankell M., Avis J., Benirschke K., Baribault H., Oshima R.G. (2003). Keratin 8 protection of placental barrier function. J. Cell Biol..

[bib16] Kalkan T., Olova N., Roode M., Mulas C., Lee H.J., Nett I., Marks H., Walker R., Stunnenberg H.G., Lilley K.S. (2017). Tracking the embryonic stem cell transition from ground state pluripotency. Development.

[bib17] Krueger F., Andrews S.R. (2011). Bismark: a flexible aligner and methylation caller for bisulfite-Seq applications. Bioinformatics.

[bib18] Leitch H.G., McEwen K.R., Turp A., Encheva V., Carroll T., Grabole N., Mansfield W., Nashun B., Knezovich J.G., Smith A. (2013). Naive pluripotency is associated with global DNA hypomethylation. Nat. Struct. Mol. Biol..

[bib19] Li J., Ran C., Li E., Gordon F., Comstock G., Siddiqui H., Cleghorn W., Chen H.Z., Kornacker K., Liu C.G. (2008). Synergistic function of E2F7 and E2F8 is essential for cell survival and embryonic development. Dev. Cell.

[bib20] Loh K.M., Lim B. (2011). A precarious balance: pluripotency factors as lineage specifiers. Cell Stem Cell.

[bib21] Martin G.R. (1981). Isolation of a pluripotent cell line from early mouse embryos cultured in medium conditioned by teratocarcinoma stem cells. Proc. Natl. Acad. Sci. U S A.

[bib22] Nichols J., Smith A. (2009). Naive and primed pluripotent states. Cell Stem Cell.

[bib23] Ouseph M.M., Li J., Chen H.Z., Pecot T., Wenzel P., Thompson J.C., Comstock G., Chokshi V., Byrne M., Forde B. (2012). Atypical E2F repressors and activators coordinate placental development. Dev. Cell.

[bib24] Pilon N., Oh K., Sylvestre J.R., Savory J.G., Lohnes D. (2007). Wnt signaling is a key mediator of Cdx1 expression in vivo. Development.

[bib25] Robinson M.D., McCarthy D.J., Smyth G.K. (2010). edgeR: a Bioconductor package for differential expression analysis of digital gene expression data. Bioinformatics.

[bib26] Shen X., Liu Y., Hsu Y.J., Fujiwara Y., Kim J., Mao X., Yuan G.C., Orkin S.H. (2008). EZH1 mediates methylation on histone H3 lysine 27 and complements EZH2 in maintaining stem cell identity and executing pluripotency. Mol. Cell.

[bib27] Smith A.G., Heath J.K., Donaldson D.D., Wong G.G., Moreau J., Stahl M., Rogers D. (1988). Inhibition of pluripotential embryonic stem cell differentiation by purified polypeptides. Nature.

[bib28] Stadler M.B., Murr R., Burger L., Ivanek R., Lienert F., Scholer A., van Nimwegen E., Wirbelauer C., Oakeley E.J., Gaidatzis D. (2011). DNA-binding factors shape the mouse methylome at distal regulatory regions. Nature.

[bib29] Ter Huurne M., Chappell J., Dalton S., Stunnenberg H.G. (2017). Distinct cell-cycle control in two different states of mouse pluripotency. Cell Stem Cell.

[bib30] Tesar P.J., Chenoweth J.G., Brook F.A., Davies T.J., Evans E.P., Mack D.L., Gardner R.L., McKay R.D. (2007). New cell lines from mouse epiblast share defining features with human embryonic stem cells. Nature.

[bib31] Trapnell C., Pachter L., Salzberg S.L. (2009). TopHat: discovering splice junctions with RNA-Seq. Bioinformatics.

[bib32] Tsanov K.M., Pearson D.S., Wu Z., Han A., Triboulet R., Seligson M.T., Powers J.T., Osborne J.K., Kane S., Gygi S.P. (2017). LIN28 phosphorylation by MAPK/ERK couples signalling to the post-transcriptional control of pluripotency. Nat. Cell Biol..

[bib33] Vallier L., Alexander M., Pedersen R.A. (2005). Activin/nodal and FGF pathways cooperate to maintain pluripotency of human embryonic stem cells. J. Cell Sci..

[bib34] Williams R.L., Hilton D.J., Pease S., Willson T.A., Stewart C.L., Gearing D.P., Wagner E.F., Metcalf D., Nicola N.A., Gough N.M. (1988). Myeloid leukaemia inhibitory factor maintains the developmental potential of embryonic stem cells. Nature.

[bib35] Xie W., Barr C.L., Kim A., Yue F., Lee A.Y., Eubanks J., Dempster E.L., Ren B. (2012). Base-resolution analyses of sequence and parent-of-origin dependent DNA methylation in the mouse genome. Cell.

[bib36] Yagi M., Kishigami S., Tanaka A., Semi K., Mizutani E., Wakayama S., Wakayama T., Yamamoto T., Yamada Y. (2017). Derivation of ground-state female ES cells maintaining gamete-derived DNA methylation. Nature.

[bib37] Yamaji M., Ueda J., Hayashi K., Ohta H., Yabuta Y., Kurimoto K., Nakato R., Yamada Y., Shirahige K., Saitou M. (2013). PRDM14 ensures naive pluripotency through dual regulation of signaling and epigenetic pathways in mouse embryonic stem cells. Cell Stem Cell.

[bib38] Ye S., Zhang D., Cheng F., Wilson D., Mackay J., He K., Ban Q., Lv F., Huang S., Liu D. (2016). Wnt/beta-catenin and LIF-Stat3 signaling pathways converge on Sp5 to promote mouse embryonic stem cell self-renewal. J. Cell Sci..

[bib39] Ying Q.L., Nichols J., Chambers I., Smith A. (2003). BMP induction of Id proteins suppresses differentiation and sustains embryonic stem cell self-renewal in collaboration with STAT3. Cell.

[bib40] Ying Q.L., Wray J., Nichols J., Batlle-Morera L., Doble B., Woodgett J., Cohen P., Smith A. (2008). The ground state of embryonic stem cell self-renewal. Nature.

[bib41] Yoshimizu T., Sugiyama N., De Felice M., Yeom Y.I., Ohbo K., Masuko K., Obinata M., Abe K., Scholer H.R., Matsui Y. (1999). Germline-specific expression of the Oct-4/green fluorescent protein (GFP) transgene in mice. Dev. Growth Differ..

