# Supplementary material for: Activin A and BMP4 Signaling Expands Potency of Mouse Embryonic Stem Cells in Serum-Free Media
Source: Stem Cell Reports. 2020 Feb 6;14(2):241–55. doi: 10.1016/j.stemcr.2020.01.004 (PMC7013251; doi:10.1016/j.stemcr.2020.01.004)
Supplement: Document S1. Supplemental Experimental Procedures and Figures S1–S6 [file mmc1.pdf]

Stem Cell Reports, Volume *14*

Supplemental Information

Activin A and BMP4 Signaling Expands Potency of Mouse Embryonic Stem Cells in Serum-Free Media

Baojiang Wu, Lin Li, Bojiang Li, Junpeng Gao, Yanglin Chen, Mengyi Wei, Zhiqing Yang, Baojing Zhang, Shudong Li, Kexin Li, Changshan Wang, M. Azim Surani, Xihe Li, Fuchou Tang, and Siqin Bao

Inventory of Supplemental Information

Supplemental Figures and Legends

Figure S1. Pluripotency of esASCs and epiASCs. Related to Figure 1

Figure S2. Molecular Features of esASCs and epiASCs. Related to Figure 2

Figure S3. Developmental Potency of esASCs and epiASCs. Related to Figure 3

Figure S4. The Signaling Pathway of ASCs. Related to Figure 4

Figure S5. Global Upregulation of DNA Methylation Level in esASCs and epiASCs. Related to Figure 5

Figure S6. The Immunostaining and Molecular Features of blASCs. Related to Figure 6

Supplemental Spreadsheets Tables

Table S1. WGBS Data Coverage and Conversion Efficiency, Related to Figure 5A

Table S2. Differential Expressed Genes and Difference in Promoter Methylation between EpiSCs and epiASCs, Related to Figure 5E

Table S3. RT-qPCR Primers, Related to Figures S4B and 5B

Supplemental Experimental Procedures

Supplemental reference

Figure S1

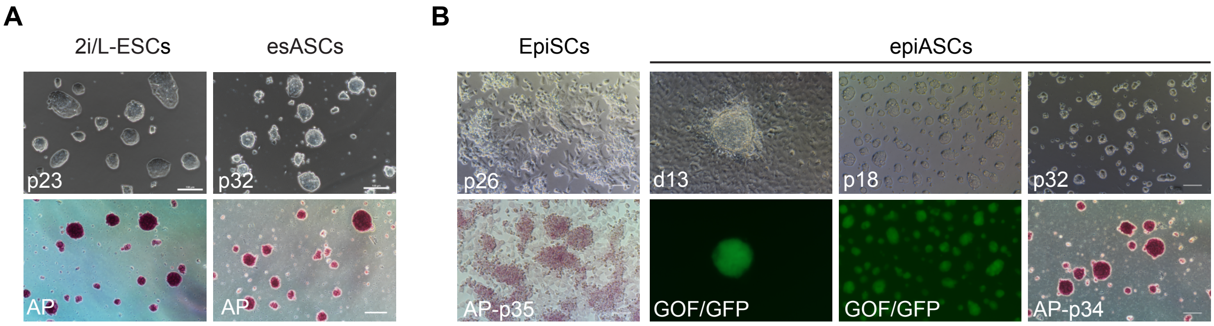

C

Name of cell lines	Cell line	Derived epiASCs	Repeat times
E6.5-1	EpiSCs	no	2
E6.5-2	EpiSCs	yes	3
E6.5-6	EpiSCs	no	3
E6.5-7	EpiSCs	no	2
E6.5-8	EpiSCs	yes	1
Total	EpiSCs	2/5 (40%)	11

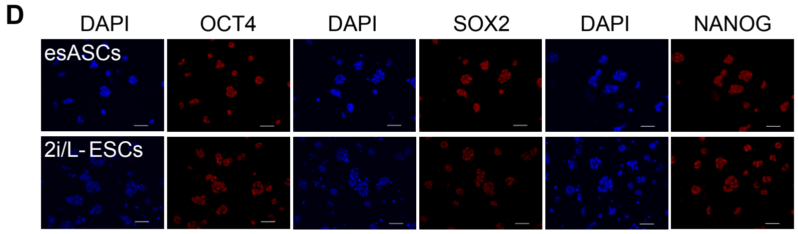

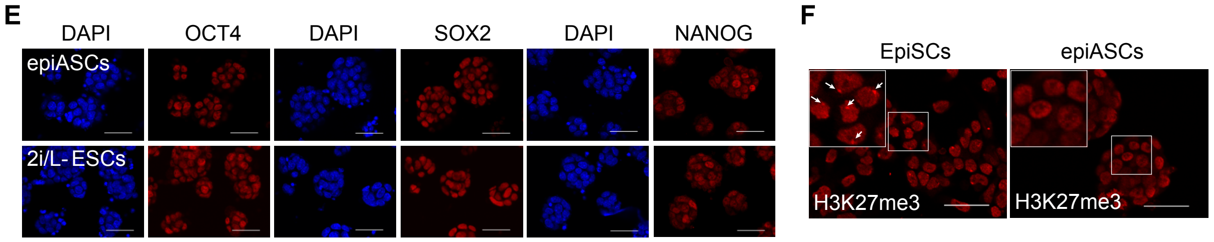

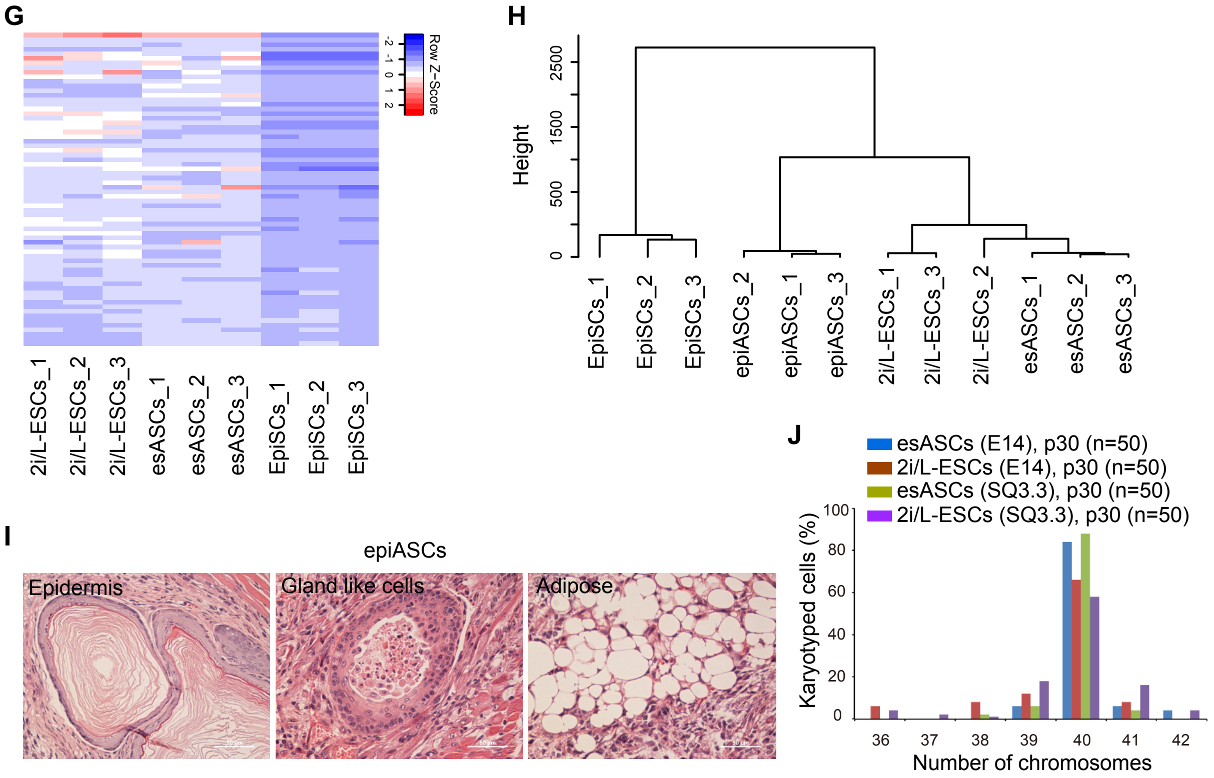

Figure S2

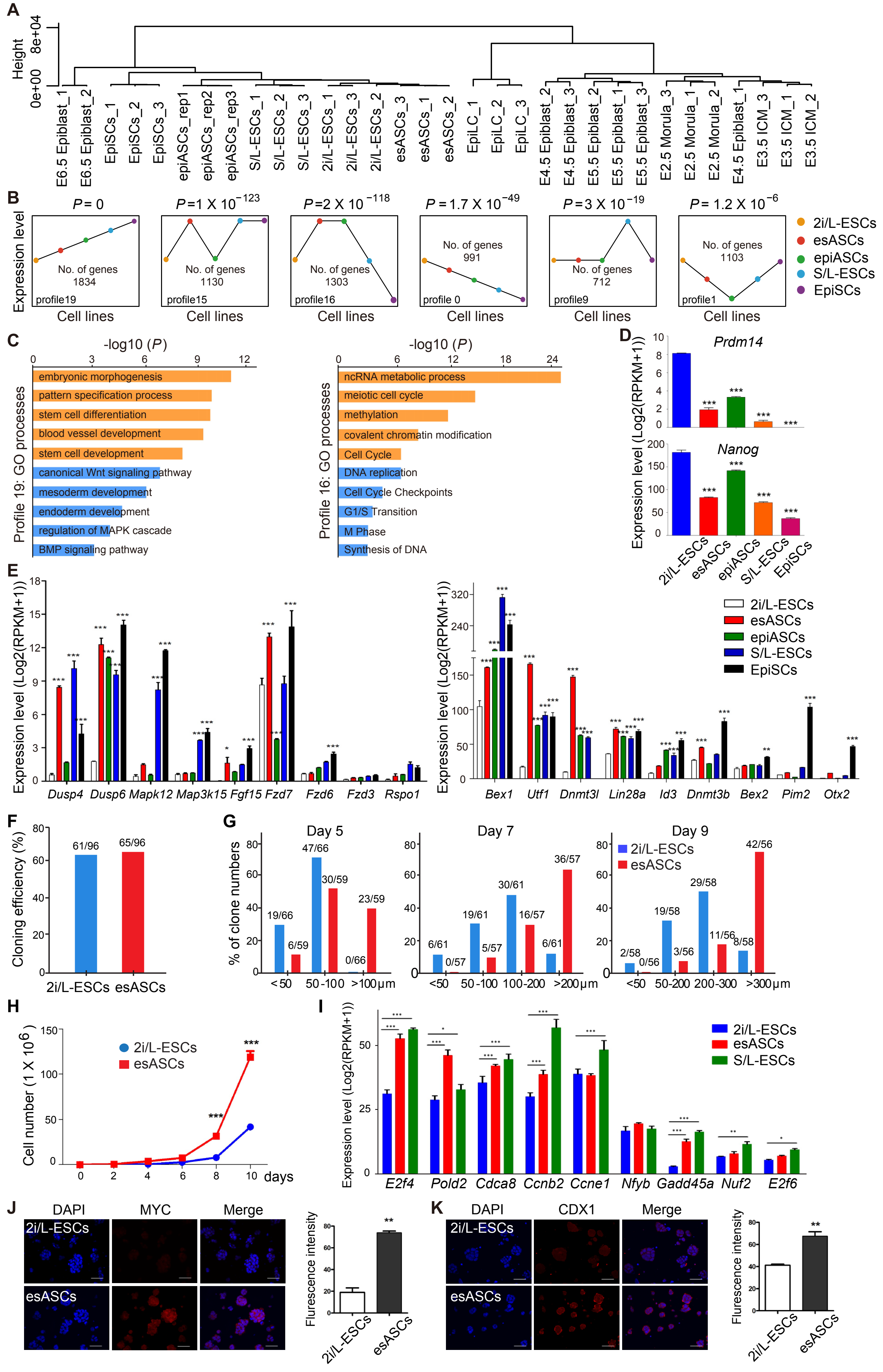

A
Height
B
Expression level
Cell lines
2i/L-ESCs
esASCs
epiASCs
S/L-ESCs
EpiSCs
C
Profile 19: GO processes
embryonic morphogenesis
pattern specification process
stem cell differentiation
blood vessel development
stem cell development
canonical Wnt signaling pathway
mesoderm development
endoderm development
regulation of MAPK cascade
BMP signaling pathway
Profile 16: GO processes
ncRNA metabolic process
meiotic cell cycle
methylation
covalent chromatin modification
Cell Cycle
DNA replication
Cell Cycle Checkpoints
G1/S Transition
M Phase
Synthesis of DNA
D
Prdm14
Nanog
Expression level (Log2(RPKM+1))
E
F
Cloning efficiency (%)
G
% of clone numbers
Day 5
Day 7
Day 9
H
Cell number (1 X 10^6)
days
I
J
DAPI
MYC
Merge
Fluorescence intensity
K
CDX1
Flurescence intensity

Figure S3

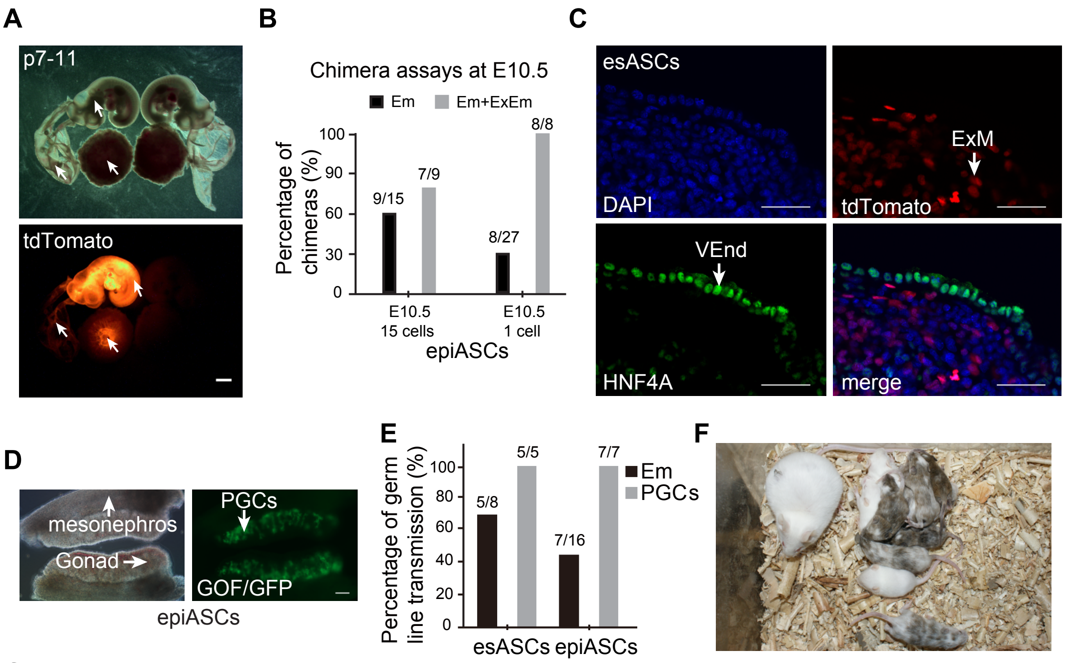

G

Stage of Embryo	Cell line	No. of injected cell	No. of Transferred embryos	No. of pups	No. of chimera	No. of ♂,♀
Full term	epiASCs	15	39	27 (69%)	20 (74%)	15, 5
Full term	epiASCs	1	53	19 (36%)	3 (16%)	1, 2
Full term	esASCs	1	20	7 (35%)	4 (57%)	2, 2
Total			112	53 (47%)	27 (51%)	18, 9

Figure S4

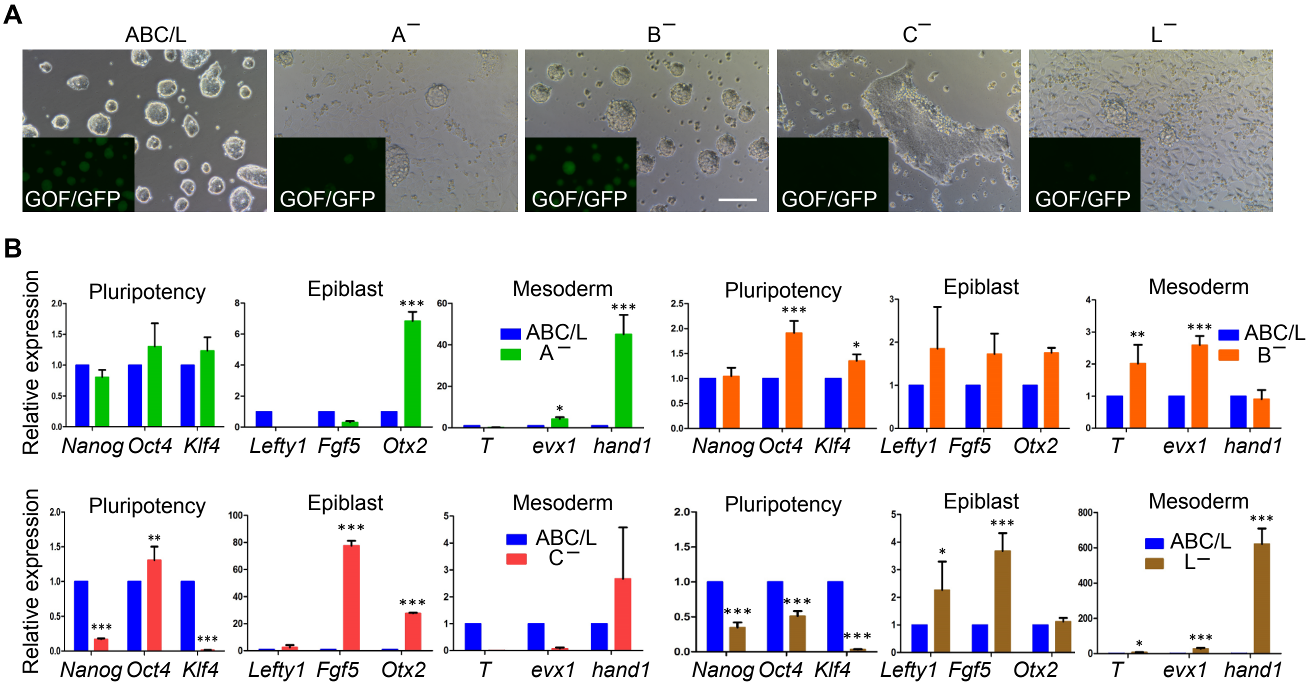
A
ABC/L
A⁻
B⁻
C⁻
L⁻
GOF/GFP
B
Relative expression
Pluripotency
Epiblast
Mesoderm
ABC/L
A⁻
B⁻
C⁻
L⁻
Nanog Oct4 Klf4
Lefty1 Fgf5 Otx2
T evx1 hand1

Figure S5

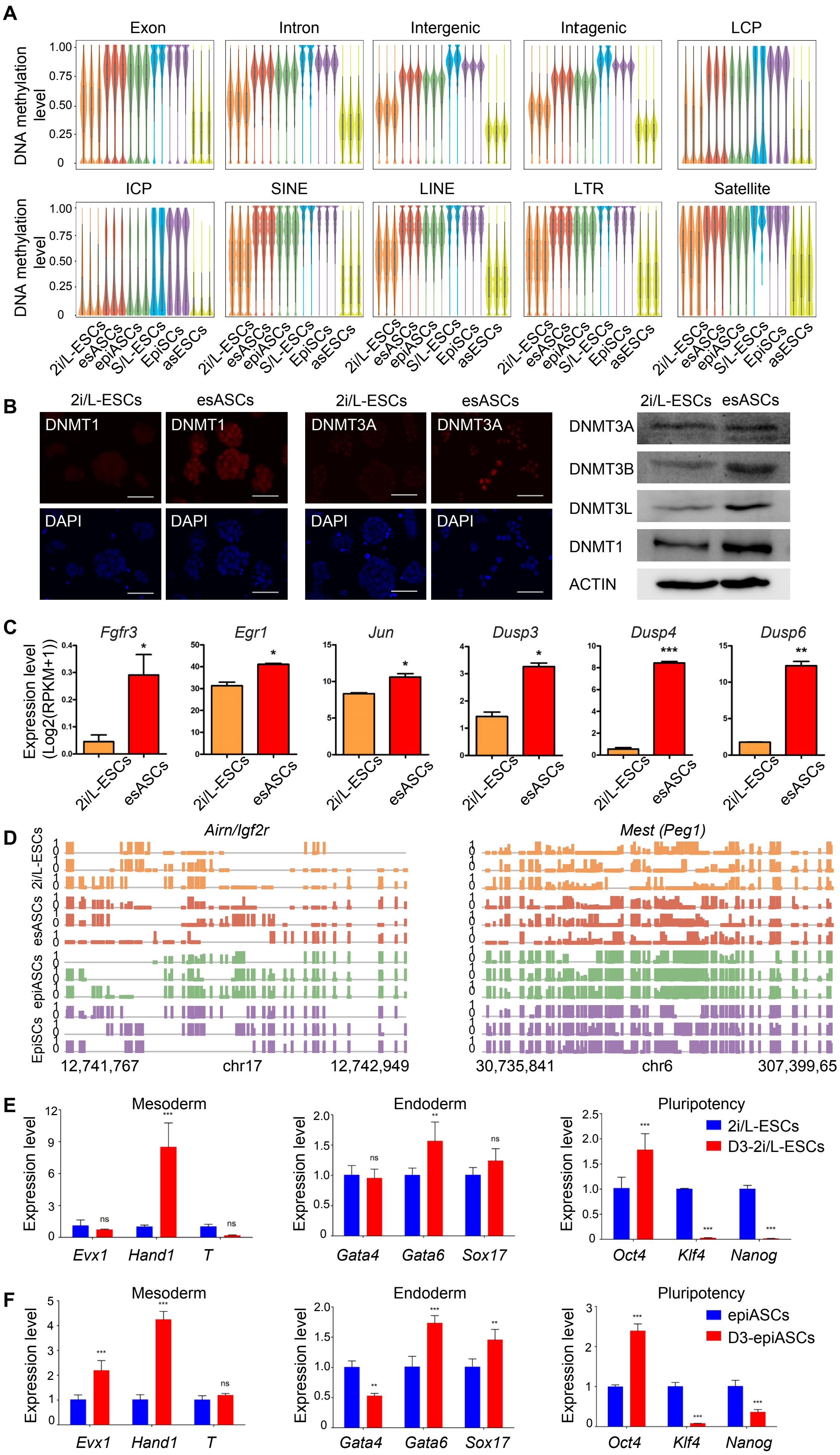
A
Exon
Intron
Intergenic
Intagenic
LCP
ICP
SINE
LINE
LTR
Satellite
DNA methylation level
2i/L-ESCs
esASCs
epiASCs
S/L-ESCs
EpiSCs
asESCs
B
2i/L-ESCs
esASCs
DNMT1
DNMT3A
DAPI
DNMT3B
DNMT3L
ACTIN
C
Fgfr3
Egr1
Jun
Dusp3
Dusp4
Dusp6
Expression level (Log2(RPKM+1))
D
Airn/Igf2r
Mest (Peg1)
12,741,767
chr17
12,742,949
30,735,841
chr6
307,399,65
E
Mesoderm
Endoderm
Pluripotency
Expression level
Evx1
Hand1
T
Gata4
Gata6
Sox17
Oct4
Klf4
Nanog
2i/L-ESCs
D3-2i/L-ESCs
F
epiASCs
D3-epiASCs

Figure S6

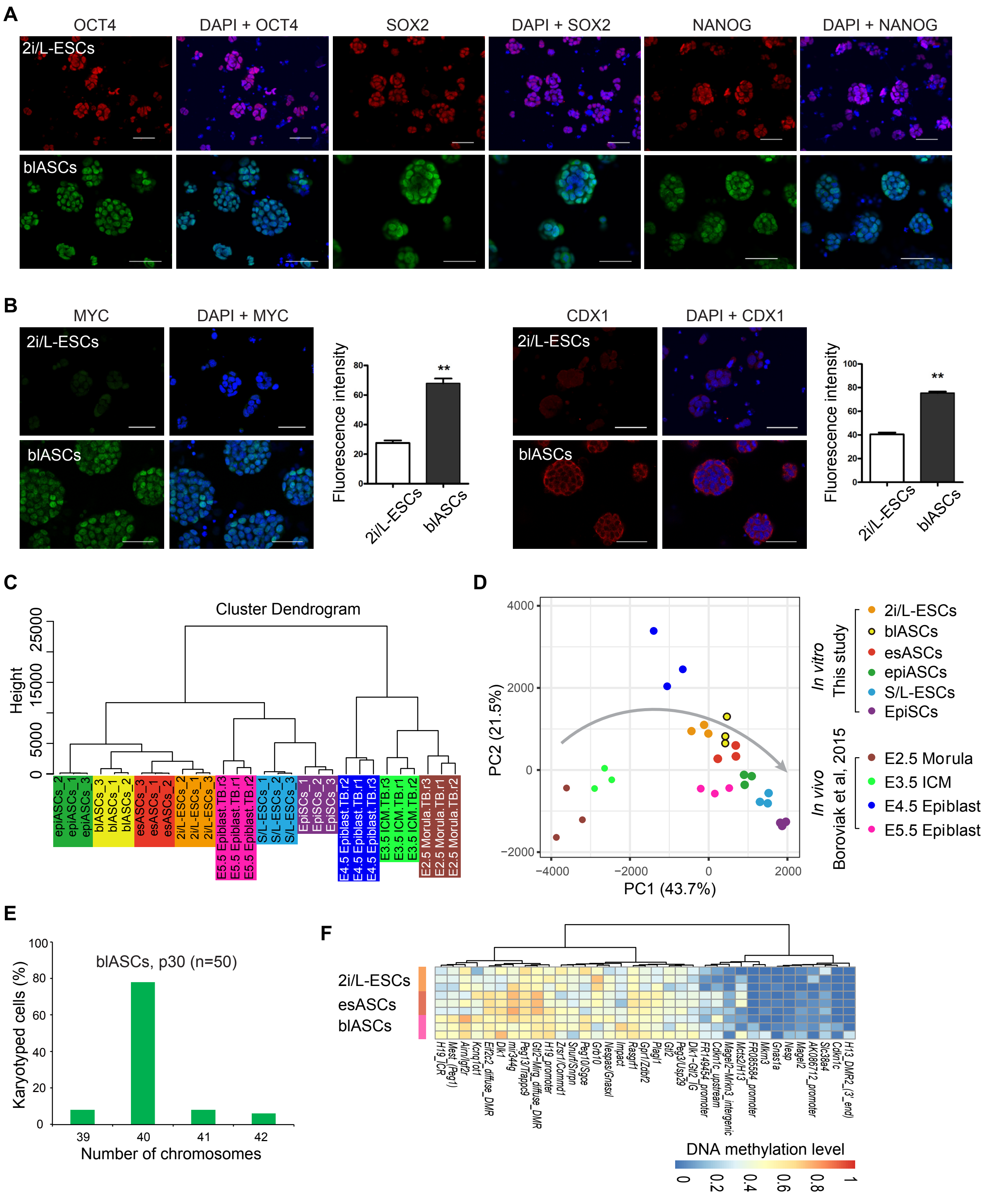

A
OCT4
DAPI + OCT4
SOX2
DAPI + SOX2
NANOG
DAPI + NANOG
2i/L-ESCs
blASCs
B
MYC
DAPI + MYC
CDX1
DAPI + CDX1
2i/L-ESCs
blASCs
Fluorescence intensity
**
C
Cluster Dendrogram
Height
0
5000
15000
25000
epiASCs_2
epiASCs_1
epiASCs_3
blASCs_3
blASCs_1
blASCs_2
esASCs_3
esASCs_1
esASCs_2
2i/L-ESCs_2
2i/L-ESCs_1
2i/L-ESCs_3
E5.5 Epiblast.TB.r3
E5.5 Epiblast.TB.r1
E5.5 Epiblast.TB.r2
S/L-ESCs_1
S/L-ESCs_2
S/L-ESCs_3
EpiSCs_1
EpiSCs_2
EpiSCs_3
E4.5 Epiblast.TB.r2
E4.5 Epiblast.TB.r1
E4.5 Epiblast.TB.r3
E3.5 ICM.TB.r3
E3.5 ICM.TB.r1
E3.5 ICM.TB.r2
E2.5 Morula.TB.r3
E2.5 Morula.TB.r1
E2.5 Morula.TB.r2
D
PC2 (21.5%)
PC1 (43.7%)
4000
2000
0
−2000
−4000
In vitro
This study
2i/L-ESCs
blASCs
esASCs
epiASCs
S/L-ESCs
EpiSCs
In vivo
Boroviak et al, 2015
E2.5 Morula
E3.5 ICM
E4.5 Epiblast
E5.5 Epiblast
E
blASCs, p30 (n=50)
Karyotyped cells (%)
0
20
40
60
80
100
39
40
41
42
Number of chromosomes
F
2i/L-ESCs
esASCs
blASCs
DNA methylation level
0
0.2
0.4
0.6
0.8
1

Supplemental Figure Legends

Figure S1. Pluripotency of esASCs and epiASCs. Related to Figure 1.

(A) Alkaline phosphatase (AP) staining on 2i/L-ESCs and esASCs. Scale bars, 100 μm.

(B) Derivation of epiASCs from EpiSCs (with GOF/GFP reporter) cultured in ABC/L medium. We started to detected GFP-positive clones in 10 days, which were able to self-renew more than 30 passages. EpiSCs indicated weak alkaline phosphatase (AP) activities; epiASCs indicated strong AP activities. Scale bars, 100 μm.

(C) Derivation rate of epiASCs from EpiSCs.

(D and E) Immunostaining of OCT4, SOX2 and NANOG in 2i/L-ESCs, esASCs and epiASCs. Scale bars, 40 μm.

(F) One X-chromosome was inactivated in female EpiSCs, and reactivated in epiASCs confirmed by lack of H3K27me3 spot. Scale bars, 40μm.

(G) RNA-Seq data indicated that X-transcripts upregulated in 2i/L-ESCs were significantly upregulated in that of esASCs and epiASCs. In addition, an unbiased hierarchical clustering analysis on X-transcripts showed that esASCs and epiASCs were closed to 2i/L-ESCs

(H) Unsupervised hierarchical clustering (UHC) of the X-transcripts from three biological replicates of four stem cell lines. Note that ASCs were clustered close to 2i/L-ESCs.

(I) Mature teratomas from epiASCs. Left: ectoderm, epidermis-like structures; middle: endoderm, gland-like cells; right: mesoderm, adipose-like structures. The sections were stained with haematoxylineosin. Scale bars, 50μm.

(J) Distribution of chromosome numbers in 2i/L-ESCs (p30) and ASCs (p30). n (n=50), number of spread analyzed and obtained from 2 independent experiments.

Figure S2. Molecular Features of esASCs and epiASCs. Related to Figure 2.

(A) Unsupervised hierarchical clustering (UHC) of the transcriptome from two to three biological replicates of five stem cell lines, EpiLC and *in vivo* E2.5-E6.5 embryos.

(B) Among differentially expressed genes, 1,834 genes (profile 19) were gradually increased followed by 2i/L-ESCs, esASCs, epiASCs, S/L-ESCs and EpiSCs, and a total of 1,303 genes (profile 16) were significantly upregulated in esASCs and epiASCs, compared with 2i/L-ESCs, S/L-ESCs and EpiSCs.

(C) The top representative GO terms (biological process) for profile 19 and 16 genes.

(D and E) Expression levels (Log2(RPKM+1)) of *Prdm 14*, *Nanog* and transcription factors, signal pathways regulating genes and epigenetic modification associated genes on five different stem cell lines. Error bars indicate SEM (n=3). Results were obtained from 3 independent experiments. The significance of differences was measured by two-way ANOVA was employed. A value of $p < 0.05$ was considered significant.

(F) Single cell clonogenicity efficiency in 2i/L-ESCs and epiASCs. Results were obtained from 2 independent experiments.

(G) The diameter of single cell formed clones were measured on day 5, 7 and 9. Results were obtained from 2 independent experiments.

(H) Cell proliferation test in 2i/L-ESCs and epiASCs. Results were obtained from 3 independent experiments. *P* values were calculated by two-way ANOVA, $p < 0.05$.

(I) Expression levels (Log2(RPKM+1)) of cell cycle associated genes in three different stem cell lines. Error bars indicate SEM (n=3). Results were obtained from 3 independent experiments. The significance of differences was measured by two-way ANOVA was employed. A value of $p < 0.05$ was considered significant.

(J and K) Immunostaining of MYC and CDX1. Fluorescence intensity was measured from 3 independent experiments, and significance was tested using the unpaired Student's *t*-test, $p < 0.05$. Scale bars, 50 μm.

Figure S3. Developmental Potency of esASCs and epiASCs. Related to Figure 3.

(A) Single epiASCs ($tdTomato^+/GOF^+$) contributed to embryo, yolk sac, and placental labyrinth in E10.5 chimeras (white arrow). Negative control (right). Scale bars, 1mm.

(B) Summary of E10.5 chimera assays by multiple and single cell injection. The black bar chart shows the percentages of chimeras among the collected E10.5 conceptuses, embryonic tissues (Em); gray bar, integration into both embryonic and extraembryonic mesoderm (Em + ExEm) among the recovered E10.5 chimeras. Results were obtained from 2 independent experiments.

(C) Contribution of esASCs (tdTomato) in chimeric yolk sac. Immunostaining of E10.5 chimeric yolk sac, extraembryonic endoderm stained by HNF4 ALPHA (green), esASCs

(tdTomato) only found in the extraembryonic mesoderm (red). Scale bars, 50 μm. ExM, extraembryonic mesoderm; VEnd, visceral endoderm.

(D) Germline transmission of epiASCs. The gonad of E12.5 chimeras shown by GOF/GFP-positive cells (arrow). Scale bars, 100 μm.

(E) Summary of E12.5 chimera assays by single esASCs and epiASCs injection. The black bar chart shows the percentages of chimeras among the collected E12.5 conceptuses, embryonic tissues (Em); gray bar, integration into primordial germ cells (PGCs) among the recovered E12.5 chimeras. Results were obtained from 2 independent experiments.

(F) Chimeric pups generated by injecting epiASCs in ICR host blastocysts.

(G) The summary of full term chimeric pups were derived by esASCs and epiASCs lines.

Figure S4. The Signaling Pathway of ASCs. Related to Figure 4.

(A) Representative images of epiASCs at 6-days inhibitor treatments. Scale bars, 100 μm.

(B) Representative gene expression levels were measured by qPCR in 6-days inhibitor treatments and control groups. Error bars indicate SEM (n=3). Results were obtained from 3 independent experiments. *P* values were calculated by two-way ANOVA, $p < 0.05$.

Figure S5. Global Upregulation of DNA Methylation Level in esASCs and epiASCs. Related to Figure 5.

(A) Violin plot showing DNA methylation level at various genomic features.

(B) Left: Immunostaining of DNMT1 and DNMT3A in 2i/L-ESCs and esASCs. Scale bars, 50 μm. Right : Western blotting analysis for DNMT3A, DNMT3B, DNMT3L and DNMT1 in 2i/L-ESCs and esASCs.

(C) Expression levels (Log2(RPKM+1)) of regulators of MAPK-ERK signaling associated genes. Error bars indicate SEM (n=3). Results were obtained from 3 independent experiments. *P* values were calculated by unpaired Student's *t*-test, $p < 0.05$.

(D) DNA methylation level of imprinted DMRs of *Airn/Igf2r* and *Mest* (*Peg1*) at four different stem cells are shown at single-base resolution. Each bar represents a methylation percentage for each CpG site.

(E) Mesoderm, endoderm and pluripotency associated genes were tested by qPCR, 2i/L-ESCs

cells after 3 days induction and control 2i/L-ESCs. Error bars indicate SEM (n=3). Results were obtained from 3 independent experiments. *P* values were calculated by two-way ANOVA, $p < 0.05$.

(F) Mesoderm, endoderm and pluripotency associated genes were tested by qPCR, epiASCs cells after 3 days induction and control epiASCs. Error bars indicate SEM (n=3). Results were obtained from 3 independent experiments. *P* values were calculated by two-way ANOVA, $p < 0.05$.

Figure S6. The Immunostaining and Molecular Features of blASCs. Related to Figure 6.

(A) Immunostaining of OCT4, SOX2 and NANOG in 2i/L-ESCs and blASCs. Scale bars, 50 μm.

(B) Immunostaining of MYC and CDX1 in 2i/L-ESCs and blASCs. Fluorescence intensity was measured from 3 independent experiments, and significance was tested using the unpaired Student's *t*-test, $p < 0.05$. Scale bars, 50 μm.

(C) Unsupervised hierarchical clustering (UHC) of gene expression profiles of pluripotent stem cells.

(D) PCA analysis of based on 1,685 dynamically expressed genes. Arrow indicates developmental progression from E2.5 morula to E5.5 postimplantation epiblast.

(E) Distribution of chromosome number in blASCs (p30). n (n=50), number of spread analyzed and obtained from 2 independent experiments.

(F) Heatmap showing DNA methylation level of ICRs in three different stem cells.

Supplemental Experimental Procedures

Derivation of 2i/L-ESCs

Mouse embryo blastocysts (E3.5) were isolated from 129/sv females mated with GOF/GFP transgenic males. Green fluorescence indicated that GFP expression of the reporter is under the control of *Oct4* promoter and distal enhancer. This GFP transgene shows expression in the ICM of blastocysts and PGC *in vivo*, and in ESCs (Yoshimizu et al., 1999). ESCs culture medium consists of N2B27 medium (Life technology) supplemented with PD0325901 (PD, 1 μM, Miltenyi Biotec), CHIR99021 (CH, 3 μM, Miltenyi Biotec) and leukemia inhibitory factor (LIF, 1000 IU/ml, Millipore), which henceforth were called 2i/L medium. Zona pellucida of blastocysts were removed by Acidic Tyrode's Solution (Sigma-Aldrich), and then placed to 24-well fibronectin-coated (FN, 16.7 μg/ml, Millipore) plate with 2i/L medium. ICM of blastocysts cultures grew efficiently and formed outgrowing colonies in 5-7 days culture. The resulting colonies were further cutting into smaller pieces by glass needles after 5-7 days culture, and then the colonies passaged by Accutase (Life technology) regularly on at every 2 days interval.

Derivation of EpiSCs

EpiSCs were derived from E6.5 epiblasts, epiblasts were cut into smaller pieces and placed to 24-well fibronectin-coated plate with N2B27 medium containing human Activin A (20 ng/ml, R&D systems) and bFGF (12 ng/ml, R&D systems). After 5-6 days culture, formed 'flat' epithelial-like colonies were cutting into smaller pieces by glass needles, and moved to new plates for further culture. After 5-7 days culture, the colonies could be passaged by Accutase (Life technology) regularly on at every 2-3 days.

Derivation of esASCs and epiASCs

2i/L-ESCs were switched on ABC/L medium which are N2B27 medium supplemented with Activin A (ActA, 20 ng/ml, R&D systems), BMP4 (50 ng/ml, R&D systems), CHIR99021 (3 μM, Miltenyi Biotec) and leukemia inhibitory factor (1000 IU/ml, Millipore), and passage every other day and we call these cells as esASCs.

We dissociated GOF/GFP EpiSCs using Accutase and placed EpiSCs (3×10^5 single cells) in fibronectin-coated 24-well plate with 1 ml ABC/L medium. Dependent on cell growth, EpiSCs were then passaged one time after 2-4 days. In 6-7 days of ABC/L treatment, we noted that the clusters induced from EpiSCs dead. GOF/GFP positive clones started to appear around day 10-14. When these GFP-positive colonies grew to around 200 μm diameter, they were picked and cut into smaller pieces using glass needle and transferred to new plate. When these colonies had grown for 6-7 days, they were treated with Accutaes, and the resulting cells were cultured to produce GFP-positive colonies, which were capable of self-renewal for over 30 passages and we call these cells as epiASCs. For inhibitor treatment experiment, we added SB431542 (10 μM, R＆D systems), Noggin (200 ng/ml, R＆D systems), XAV939 (10 μM, Sigma-Aldrich), JAK inhibitor I (0.6 μM, Calbiochem) into ASCs culture medium.

Derivation of S/L-ESCs

2i/L-ESCs were switched to fibronectin-coated plate with standard ES medium (Knockout DMEM; Knockout Dulbecco's modified Eagle's medium) supplemented with 20% fetal calf serum, 0.1 mM 2-mercaptoethanol, 2 mM L-glutamine, 0.1 mM non-essential amino acid, 50 U/ml Penicillin/Streptomycin and 1000U/ml LIF without feeder cells, we named these cells as S/L-ESCs.

Cell Differentiation

2i/L-ESCs and epiASCs were cultured in N2B27 medium for 3 days withdrawal of PD, CH and LIF, and ActA, BMP4, CH and LIF respectively.

Colony Formation Assay

Single 2i/L-ESCs and epiASCs were seeded at a fibronectin-coated 96-well plates using mouth pipette, containing 2i/L and ABC/L medium, respectively. The cells were cultured for 10 days and the number of colonies was assessed.

Flow Cytometry

ESCs were harvested by Accutaes and fixed 30min in 3.7% PFA at room temperature. After

fixing, cells were incubated with 15 μg/ml Hoechst 33342 (Invitrogen) for 1 hour at 37℃ and analyzed on the BD LSRFortessa. Data analysis was performed using FlowJo software (Ashland). Samples of at least 10,000 cells (GOF/GFP^+) were acquired using a BD LSRFortessa flow cytometre (Becton Dickinson). Green fluorescence indicated that GFP expression of the reporter is under the control of *Oct4* promoter and distal enhancer. This GFP transgene shows expression in the ICM of blastocysts and PGCs *in vivo*, and in ESCs (Yoshimizu et al., 1999). Hoechst 33342 untreated ESCs were used for FACS gating negative control. Measure fluorescence (detector 488 nm channel for GFP and 355nm channel for Hoechst 33342) by flow cytometer. Gating out of residual cell debris and measure diploid and tetraploid DNA peaks. Identification of G1, S and G2/M phase by Hoechst 33342. The histogram of DNA content could categorize cells into three groups, G1, S and G2/M phase. The final percentage (%) of cells for each phase is about G1, S, G2/M according to the analysis. First create a dot plot displaying forward scatter (FSC) on a linear scale and GFP on a logarithmic scale. A region representing GFP-positive cells were used to identifiy living cells. Create a second dot plot region representing side scatter (SSC) and Hoechst 33342 cells (both on a linear scale) to include all live DNA-positive cells. Create a third dot plot displaying SSC and Hoechst 33342 cells (linear scale) to set the emission for determining DNA labeling in live cells.

Derivation of blASCs Cell Lines

Mouse blastocysts (E3.5) were isolated from 129/sv females mated with Oct4-ΔPE-GFP (GOF/GFP) transgenic males. Zona pellucida of blastocysts were removed by Acidic Tyrode's Solution (Sigma-Aldrich), and then placed to 24-well fibronectin-coated (16.7 μg/ml, Millipore) plate with ABC/L medium. ICM of blastocysts cultures grew efficiently and formed outgrowing colonies in 5-7 days culture. The resulting colonies were further cutting into smaller pieces by glass needles after 5-7 days culture, and then the colonies passaged by Accutase (Life technology) regularly on at every 2 days interval. Blastocyst derived GFP-positive colonies, which were capable of self-renewal for over 30 passages. We call these cells as blASCs.

Alkaline Phosphatase (AP) Staining

AP staining was carried out using AP staining kit from Sigma (86R-1KT) according to manufacturer's instructions. Briefly, the cells were fixed by 4% paraformaldehyde for 10 min, and then were stained by AP staining solution for overnight at room temperature.

Immunostaining

Cultured cells were briefly washed with PBS and fixed in 4% paraformaldehyde in PBS for 15 min at room temperature. Cells were permeabilized for 30 min with 1% BSA and 0.1% Triton X-100 in PBS. Antibody staining was carried out in the same buffer at 4 ℃ for overnight. The slides were subsequently washed three times in 1% BSA, 0.1% Triton X-100 in PBS (5 min each wash), were incubated with secondary antibody for 1h at room temperature in the dark, washed once for 5 min in 1% BSA, 0.1% Triton X-100 in PBS and twice for 5 min in PBS. The slides were then mounted in Vectashield with DAPI (Vector Laboratories) and imaged using an Olympus FV1000 confocal microscope. Primary antibodies used were: mouse monoclonal Oct4 (BD Biosciences, Catalog Number: 611203, 1:200), rat monoclonal NANOG (eBioscience, Catalog Number: 14-5761, 1:500), goat polyclonal SOX2 (Santa cruz, Catalog Number: sc-17320, 1:200), rabbit polyclonal H3K27me3 (Upstate, Catalog Number: 07-449, 1:500), MYC (Abcam, Catalog Number: ab320721:200), HNF4A (Santa cruz, Catalog Number: sc-374229, 1:100), CDX1 (Abcam, Catalog Number: ab118358, 1:200) and anti-RFP antibody (Rockland, Catalog Number: 600-401-379, 1:200). All secondary antibodies used were Alexa Fluor highly crossed adsorbed (Molecular Probes).

Teratomas Formation

The 2i/L-ESCs and epiASCs were disaggregated using Accutase, and 1×10^6 cells were injected into under epithelium of NOD–SCID mice. Three to five weeks after transplantation, tumor (s) were collected and fixed with 4% paraformaldehyde, and processed for paraffin sectioning. Sections were observed following Hematoxylinand Eosin staining.

Karyotyping

ESCs were prepared for cytogenetic analysis by treatment with colcemid (Sigma) at a final concentration of 0.1 μg/ml for 3h to accumulate cells in metaphase. Cells were then exposed to 0.075 M KCl for 25 min at 37℃ and fixed with 3:1 methanol: acetic acid. Air-dried slides were generated and G-banded following standard GTG banding protocols.

Production of Full Term Pups in Tetraploid Host Blastocysts

Two-cell stage embryos (E1.5) from ICR were collected by flushing oviducts; these were subjected to electrofusion to create tetraploid (4N) host blastocysts. Typically 15-20 ASCs were injected into tetraploid host blastocysts, which were transferred to pseudopregnant recipients at 2.5dpc, and wait for full term pups to born.

Real-Time PCR

Total RNA was isolated with the RNeasy Plus Mini Kit (Qiagen) and reverse transcribed into cDNA using the Reverse Transcription System (Promega) according to the manufacturer's instructions. Quantitative real-time PCR (qRT-PCR) was conducted using a PikoReal Real-Time PCR System (Thermo Scientific) and qRT-PCR reaction was performed with KAPA SYBR FAST qPCR kit (KAPA Biosystems). At least triplicate samples were assessed for each gene of interest, and GAPDH was used as a control gene. Relative expression levels were determined by the $2^{-\Delta\Delta Ct}$ method. Primer sequences used are given in Table S3.

Supplemental Reference

Yoshimizu, T., Sugiyama, N., De Felice, M., Yeom, Y.I., Ohbo, K., Masuko, K., Obinata, M., Abe, K., Scholer, H.R., and Matsui, Y. (1999). Germline-specific expression of the Oct-4/green fluorescent protein (GFP) transgene in mice. Dev Growth Differ *41*, 675-684.
